# Egr-1 regulates RTA transcription through a cooperative involvement of transcriptional regulators

**DOI:** 10.18632/oncotarget.20648

**Published:** 2017-09-05

**Authors:** Roni Sarkar, Subhash C. Verma

**Affiliations:** ^1^ Department of Microbiology and Immunology, University of Nevada, Reno School of Medicine, Reno, NV, USA

**Keywords:** KSHV, lytic reactivation, RTA, Egr-1, CBP

## Abstract

Kaposi’s sarcoma associated herpesvirus (KSHV) regulates the host cellular environment to establish life-long persistent infection by manipulating cellular signaling pathways, with approximately 1- 5% of cells undergoing lytic reactivation during the course of infection. Egr-1 (Early Growth Response Factor-1) is one such cellular transcription factor, which gets phosphorylated during the lytic phase of viral life cycle to perpetrate its function. This study demonstrates the mechanism of how Egr-1 mediates transcription of the immediate early gene, RTA (Replication and transcription activator), which is the lytic switch gene of KSHV. Egr-1 depleted KSHV infected cells exhibited reduced expression of RTA. Also, an increase in Egr-1 phosphorylation led to a higher virion production, which was suppressed in the presence of p38 and Raf inhibitors. Reporter assays showed that coexpression of Egr-1 and CBP (CREB-binding protein) enhances RTA promoter activity as compared to the expression of either Egr-1 or CBP alone. Binding of Egr-1 and CBP at RTA promoter was analyzed by chromatin immunoprecipitation assay (ChIP), which showed an enhanced accumulation during viral reactivation. Mutation in Egr-1 binding site of the RTA promoter eliminated Egr-1 response on promoter activation. Furthermore, *de novo* infection of THP-1 (monocytic) and HUVECs (endothelial) cells showed an upregulation of Egr-1 phosphorylation, whereas depletion of Egr-1 reduced the mRNA levels of RTA during primary infection. Together, these results demonstrate a cooperative role of Egr-1 and CBP in mediating RTA transcription, which significantly improves our understanding of the involvement of cellular factors controlling RTA transcription in KSHV pathogenesis.

## INTRODUCTION

KSHV is associated with various human malignancies such as Multicentric Castleman’s Disease [1], Primary Effusion Lymphomas (PELs) and Kaposi’s sarcoma, with the immune-compromised individuals having higher prevalence [2, 3]. Kaposi’s sarcoma-associated herpesvirus (KSHV; also, known as human herpesvirus 8) was discovered from the KS lesions from patients with AIDS-associated Kaposi’s sarcoma in 1994 [4, 5]. The life cycle of KSHV predominantly displays latent phase with a restricted gene expression, and a lytic phase expressing all the genes and production of progeny virions. The capability to maintain a lifelong persistent infection of this virus can be attributed to its immune evasive abilities, which help escaping the host’s innate and adaptive defense mechanisms [6–8].

The viral latency in infected cells can be reversed to lytic phase by various chemical inducers and viral gene products [9, 10]. Based on their expression patterns, the genes expressed during lytic cycle have been grouped into immediate early, early, and the late phase genes [11]. The immediate early genes of viral life cycle play a significant role because of their capability to drive the transcription of various downstream genes through the involvement of transcription factors [12]. The viral lytic switch protein, RTA (replication and transcription activator) plays a crucial role in lytic reactivation [9, 13]. It not only activates the transition from latent to lytic phase, but also controls the transcription of various viral genes during the lytic phase [14–17]. During *de novo* infection, latency is usually followed by a short lytic phase with differential gene expression [18, 19]. In fact, the activation of lytic cycle and the establishment of latency depend mainly on various viral and cellular factors that regulate the expression and the activity of RTA [20]. The active role of one such cellular factor, Egr-1 has been documented in controlling RTA transcription during lytic reactivation [21, 22].

Egr-1 (zif268/ NGFI-A/ Krox24), a zinc finger DNA-binding protein, deregulates the expression of the target genes by binding to their promoters [23–27]. It regulates genes of various pathways that are involved in cellular proliferation [28, 29], differentiation [30, 31] and apoptosis [32–34]. Extracellular stimuli trigger the expression of Egr-1, to modulate various signaling cascades through alteration of target genes expression [35–37]. However, not much is known about the role of Egr-1 in the replication of KSHV and disease pathogenesis. Some studies conducted on viral infection [38–45], including KSHV [21, 22, 46] have evidenced an enhanced level of Egr-1. The involvement of Egr-1 in regulating viral genes has also been reported in the transcription of latency-associated transcripts (LATs) of HSV-1 [47].

In the context of KSHV, Egr-1 has been shown to associate with RTA promoter in the infected cells and a treatment with resveratrol suppressed viral reactivation by decreasing the levels of Egr-1 [21, 22]. Egr-1 is known to interact with transcriptional coactivators, CBP/p300 to trigger the transcription of various cellular genes [24, 48, 49]. Additionally, CBP/p300 are capable of associating with various other transcription regulators to modulate different cellular pathways by interfering at the level of transcription [48, 50, 51]. This led us to hypothesize that RTA transcription could be regulated by an interaction of Egr-1 with CBP/p300 at RTA promoter.

Here, we show that depletion of Egr-1 from KSHV infected cells leads to a reduction in virion production following lytic reactivation. Also, induction of Egr-1 phosphorylation followed by enhanced virion production have been evidenced by Okadaic acid treatment, whereas suppression of both phosphor-Egr-1 and generation of virions seemed to occur on incubating with p38 MAP kinase/Raf inhibitors. Since, Egr-1 interacts with CBP/p300, we wanted to analyze whether Egr-1 mediated RTA promoter activity could be affected by CBP/p300 co-expression. Our findings from the reporter assay confirmed a cooperative effect of CBP/p300 with Egr-1 in augmenting the RTA promoter activity. Moreover, during viral reactivation protein interaction and chromatin immunoprecipitation assays determined an enhanced binding of Egr-1 and CBP as well as an elevated association at the RTA promoter region. Depletion of Egr-1, CBP or both followed by a detection of RTA transcripts confirmed a cooperative effect of Egr-1 and CBP since cells undergoing dual gene depletion showed significant reduction in RTA mRNA. Through combined data of ChIP and reporter assays, we demonstrate that Egr-1, CBP/p300 bind at the RTA promoter to regulate its transcription. Additionally, we determined the role of Egr-1 in regulating RTA expression during primary *de novo* infection of KSHV. An analysis of RTA promoter activity during *de novo* infection of KSHV showed a reduced transcriptional activity of promoter with mutated Egr-1 site as compared to the wild type. Moreover, we also found that depletion of either Egr-1 or CBP leads to a reduction in RTA promoter activity during *de novo* infection. Altogether, these results add significant information, which will impact the understanding of Egr-1 and CBP as a potential therapeutic target for blocking KSHV lytic replication.

## RESULTS

### Egr-1 expression controls virion production during lytic reactivation

**Figure 1:**
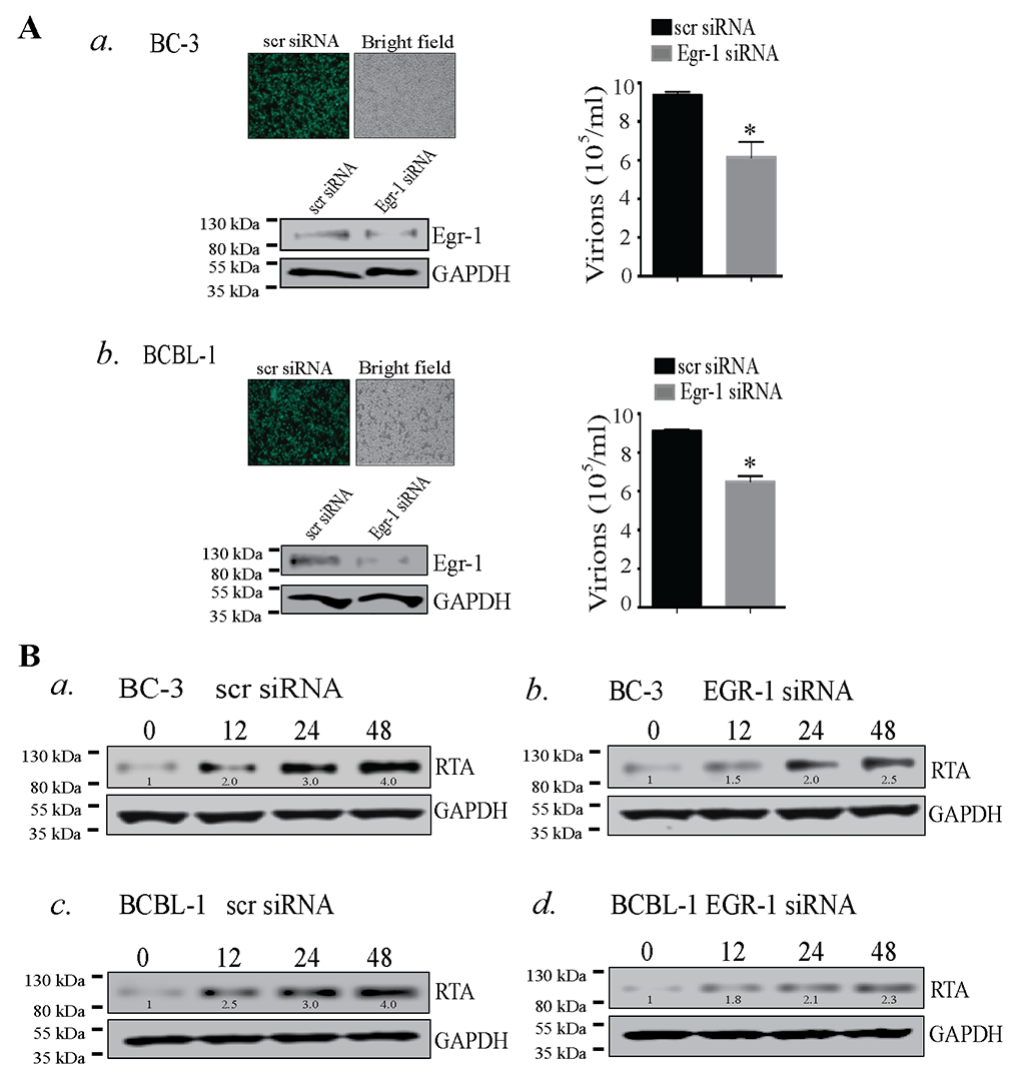
(A) Egr-1 silencing reduced virion production in BC-3 and BCBL-1 cells a. BC-3 and b. BCBL-1 cells were transfected with scrambled siRNA (scr siRNA)/Egr-1 siRNA and allowed to grow for 24h followed by induction with TPA for 72h. Culture supernatant containing virions was collected and concentrated through ultracentrifugation. The relative virion quantity was determined by qPCR of the DNA extracted from the virions. The expression of Egr-1 and the respective GAPDH are shown in BC-3 and BCBL-1 transfected with scr/ Egr-1 siRNA. Transfection efficiencies of scrambled siRNA (scr siRNA-FITC conjugated) (green cells) are shown in BC-3 and BCBL-1, respectively. ^*^P < 0.05. **(B)** RTA expression gets reduced during viral reactivation in BC-3 and BCBL-1 cells depleted of Egr-1 expression. BC-3 cells transfected with (scr/Egr-1 siRNA) were analyzed for RTA expression after induction for respective time points, 0, 12, 24 and 48h. RTA expression levels were compared between BC-3 transfected with a. scrambled siRNA (scr siRNA) and b. Egr-1 siRNA. BCBL-1 cells were analyzed for RTA expression after induction for respective time points, 0, 12, 24 and 48h. The RTA expression levels were compared between BCBL-1 transfected with c. scrambled siRNA (scr siRNA) and d. Egr-1 siRNA. The densitometric analyses of RTA expression at respective time points, 0, 12, 24 and 48h are shown in BC-3 and BCBL-1 cells transfected with scr/Egr-1 siRNA.

The lytic reactivation of KSHV is triggered by various stimuli in which RTA plays a predominant role by switching the lytic cycle cascade. The role of Egr-1 as a cellular transcription factor depicts its various functional properties such as association with other transcription factors [24, 52] and phosphorylation [53, 54], which could determine the transcriptional fate of different genes. Earlier reports evinced the involvement of Egr-1 in the expression of RTA in KSHV infected HMVEC-d cells [21], and treatment with resveratrol reduced viral reactivation via suppressing Egr-1 levels in KSHV infected cells [22]. Importantly, resveratrol is known to activate MAPK family members such as p38, ERK 1/2 and JNK [55, 56], and is involved in multiple cellular machinery with diverse biological effects [56]. Though resveratrol could have significant therapeutic value but the activation of multiple cellular cascades makes the interplay of Egr-1 and RTA obscure. Hence, a study depicting the direct mechanism of how Egr-1 regulates the expression of RTA is mandatory. To answer these questions, we used BC-3 and BCBL-1 cells, which maintain the viral genome in a latent state [57, 58], for quantifying the amounts of virions produced after Egr-1 depletion. The Egr-1 depleted cells were induced with TPA for 72h followed by quantitation of virions, which showed a significant reduction as compared to the control siRNA transfected cells (Figure 1A, *a* for BC-3 and Figure 1A, *b* for BCBL-1). Depletion of Egr-1 was confirmed by detection of total Egr-1 protein from the Egr-1 siRNA transfected cells (Figure 1A, *a* for BC-3 and Figure 1A, *b* for BCBL-1). The transfection efficiencies of scr siRNA (scrambled siRNA) in BC-3 and BCBL-1 cells are shown in Figure 1A, *a* for BC-3 and Figure 1A, *b* for BCBL-1. The expression level of RTA was also monitored at 12, 24 and 48h after induction in both scr siRNA and Egr-1 siRNA transfected cells after TPA treatment (Figure 1B). Importantly, both the KSHV positive cells, BC-3 and BCBL-1, depleted for Egr-1 showed reduced levels of RTA expression at all the time points post-induction (Figure 1B, compare panels, *b* and *d* with *a* and *c*). Thus, the suppression in RTA expression and reduced virion production form the Egr-1 depleted cells (BC-3 and BCBL-1) clearly indicate that Egr-1 regulates virion production by augmenting RTA expression.

### Phosphorylation of Egr-1 through p38 and Raf kinases is required for virion production

**Figure 2:**
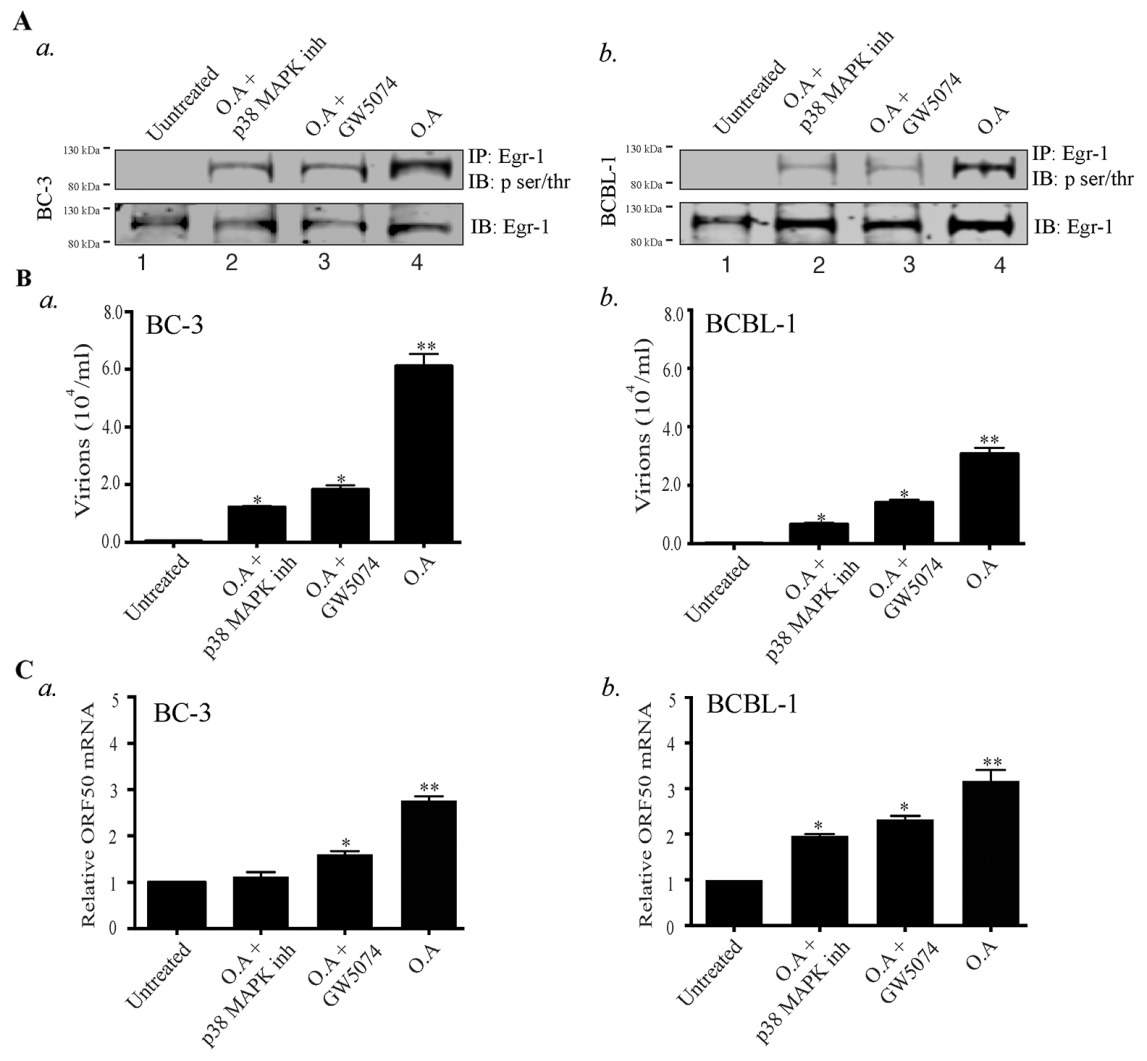
Okadaic acid (O.A) upregulates Egr-1 phosphorylation and virion production, which get suppressed by p38 MAP kinase (p38 MAPK) and Raf kinase inhibitors **(A)** a. BC-3 and b. BCBL-1 cells were treated with the combination of Okadaic acid (30 ng/ml); Okadaic acid (30 ng/ml) and p38 MAP kinase inhibitor (30 ng/ml); Okadaic acid (30 ng/ml) and GW5074 (50 µM) respectively for 24h. The cells were collected, lysed and processed for the detection of Egr-1 phosphorylation (IB: p-ser/thr) through immunoprecipitation of total Egr-1 and immunoblotting. Lane 1- Untreated, Lane 2- Okadaic acid + p38 MAP kinase inhibitor, Lane 3- Okadaic acid + GW5074, Lane 4- Okadaic acid. **(B)** Culture supernatant (containing virions) from a. BC-3 and b. BCBL-1 cells, treated with the combination of Okadaic acid (30 ng/ml); Okadaic acid (30 ng/ml) and p38 MAP kinase inhibitor (30 ng/ml); Okadaic acid (30 ng/ml) and GW5074 (50 µM) were collected after 72h and concentrated through ultracentrifugation. The concentrated virions were processed for viral genome extraction and quantification through qPCR. ^*^P < 0.05, ^**^ *P* < 0.01. **(C)** a. BC-3 and b. and BCBL-1 cells were treated with the combination of Okadaic acid (30 ng/ml); Okadaic acid (30 ng/ml) and p38 MAP kinase inhibitor (30 ng/ml); Okadaic acid (30 ng/ml) and GW5074 (50 µM) for 24h. The cells were harvested for the analysis of ORF50 transcripts through qPCR. ^*^P < 0.05, ^**^ *P* < 0.01.

The significance of Egr-1 phosphorylation in the enhancement of DNA binding activity and transcription of target genes have been established [53, 54] but information related to the importance of Egr-1 phosphorylation in viral infection is lacking. Various upstream effectors (p38/JNK, Raf/MEK/ERK) have been evidenced to be involved with Egr-1 phosphorylation [36, 59, 60]. Besides, the MEK/ERK, JNK/p38 and various MAPK pathways are known to reactivate KSHV from latency [61]. Hence, the involvement of these kinase dependent pathways in regulating Egr-1 phosphorylation cannot be ignored. Therefore, we were interested to determine whether Egr-1 phosphorylation contributes to viral reactivation and virion production. Additionally, involvement of specific kinases controlling Egr-1 phosphorylation, during lytic induction, needed to be determined for identifying the signaling pathways. To answer these key questions, we used Okadaic acid, an inducer of Egr-1 phosphorylation [62], to treat BC-3 and BCBL-1 cells, which enhanced Egr-1 phosphorylation in BC-3 and BCBL-1 cells (Figure 2A, *a*. and *b*, lane 4). In addition, we incubated the Okadaic acid treated cells with either Raf inhibitor (GW5074) or p38 MAPK inhibitor, respectively. The results indicated that the inhibitors of p38 and Raf kinases reduced the phosphorylation of Egr-1 (induced through Okadaic acid) in BC-3 and BCBL-1 cells (Figure 2A, *a* and *b*, lanes 2 and 3). Next, we determined whether the phosphorylated form of Egr-1 was involved in the regulation of transcriptional machinery during viral lytic program. We attained this by quantifying the virions produced from cells (BC-3 and BCBL-1) treated with the combination of either Okadaic acid/ Okadaic acid and p38 MAP kinase inhibitor/ Okadaic acid and GW5074, respectively. The results showed that the virions produced from the Okadaic acid treated cells (BC-3/BCBL-1) were reduced in the presence of specific inhibitors of p38MAP kinase and Raf pathways (Figure 2B, *a* for BC-3 and *b* BCBL-1). We also confirmed the expression of RTA by analyzing its mRNA levels in treated cells. The results showed that Okadaic acid treatment induced ORF50 (RTA) transcription, whereas the presence of either p38 MAP kinase inhibitor or GW5074 reduced the levels of ORF50 transcripts (Figure 2C, *a* for BC-3 and Figure 2C, *b* for BCBL-1). Taken together, these results indicated that Egr-1 phosphorylation via the involvement of p38 and Raf kinases is linked to ORF50 transcription and virion production.

### EGR-1 augments RTA promoter activity in presence of CBP/p300

**Figure 3:**
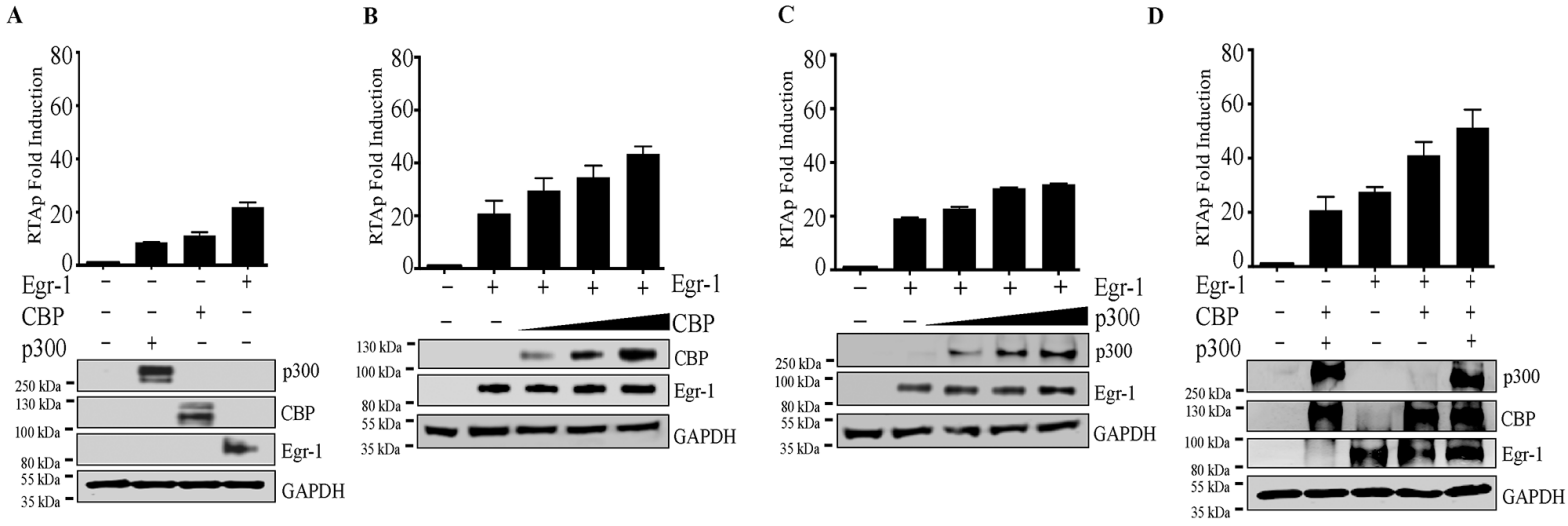
**(A)** Egr-1/CBP/p300 upregulates RTA promoter activity. A total of 2 × 10^6^ 293T cells were transfected with either RTAp (0.1µg); RTAp (0.1µg), p300 (0.5µg); RTAp (0.1µg), CBP (0.5µg); RTAp (0.1µg), Egr-1 (0.5µg). The cells were harvested after 48h post-transfection for the luciferase assay. **(B)** CBP upregulates RTA promoter (RTAp) activity in the presence of Egr-1. A total of 2 × 10^6^ 293T cells were co-transfected with the combination of either RTAp (0.1µg); RTAp (0.1µg), Egr-1 (0.5µg); RTAp (0.1µg), Egr-1 (0.5µg), CBP (0.5µg); RTAp (0.1µg), Egr-1 (0.5µg), CBP (1.0µg); RTAp (0.1µg), Egr-1 (0.5µg), CBP (1.5µg). The cells were harvested after 48h post-transfection for luciferase assay. **(C)** p300 upregulates RTA promoter activity in the presence of Egr-1. A total of 2 × 10^6^ 293T cells were co-transfected with either RTAp (0.1µg); RTAp (0.1µg), Egr-1 (0.5µg); RTAp (0.1µg), Egr-1 (0.5µg), p300 (0.5µg); RTAp (0.1µg), Egr-1 (0.5µg), p300 (1.0µg); RTAp (0.1µg), Egr-1 (0.5µg), p300 (1.5µg). The cells were harvested after 48h post-transfection for luciferase assay. **(D)** Egr-1 along with CBP and p300 upregulates RTA promoter activity. A total of 2 × 10^6^ 293T cells were transfected with either RTAp (0.1µg); RTAp (0.1µg), CBP (0.5µg), p300 (0.5µg); RTAp (0.1µg), Egr-1 (0.5µg); RTAp (0.1µg), Egr-1 (0.5µg), CBP (0.5µg); RTAp (0.1µg), Egr-1 (0.5µg), CBP (0.5µg), p300 (0.5µg). The cells were harvested after 48h post-transfection for luciferase assay.

Based on previous studies, Egr-1 interacts with CBP/p300 [24, 63], which is involved in the transcriptional regulation of different target genes [48]. In terms of KSHV, the individual expression of CBP/p300 was known to modulate RTA promoter activity [49]. However, it was not clear whether CBP/p300 act cooperatively with Egr-1 to regulate RTA transcription. Our results validated the interaction of Egr-1 with transcriptional coactivators CBP in KSHV infected BC-3 and BCBL-1 cells (Figure 5B, *a* for BC-3 and Figure 5B, *c* for BCBL-1). We conducted a luciferase reporter assay to analyze the RTA promoter activity by co-expressing CBP and p300 along with Egr-1 and analyzed the RTA promoter activity in the presence of either Egr-1, CBP or p300 expressing cells. Our results showed an activation of RTA promoter in the presence of Egr-1, CBP or p300 (Figure 3A). In order to identify the cooperative effects of CBP/p300 along with Egr-1, we co-expressed either CBP or p300 in a dose dependent manner keeping the concentration of Egr-1 constant (Figure 3B and Figure 3C). The results showed an upregulation of RTA promoter with a gradual increase in concentration of CBP and p300 (Figure 3B for CBP; Figure 3C for p300). Importantly, the cooperative effect of CBP and Egr-1 was higher as compared to the combined dosage of p300 and Egr-1 in modulating the RTA promoter (Figure 3B and Figure 3C). There observations were consistent with the previous reports of RTA upregulation by either CBP or p300 [49]. To verify the cooperative effects of CBP and p300 with Egr-1 on RTA promoter activation, we co-expressed CBP and p300 with and without Egr-1, respectively (Figure 3D). Interestingly, we found that both CBP and p300 cooperatively activate RTA promoter in the absence of Egr-1. However, expression of Egr-1 significantly activated RTA promoter, confirming that Egr-1 can augment the transcriptional activities of CBP/p300 (Figure 3D). Altogether, the reporter assay data confirmed that RTA promoter activity gets regulated through Egr-1 by the cooperative effect of CBP/p300.

**Figure 4:**
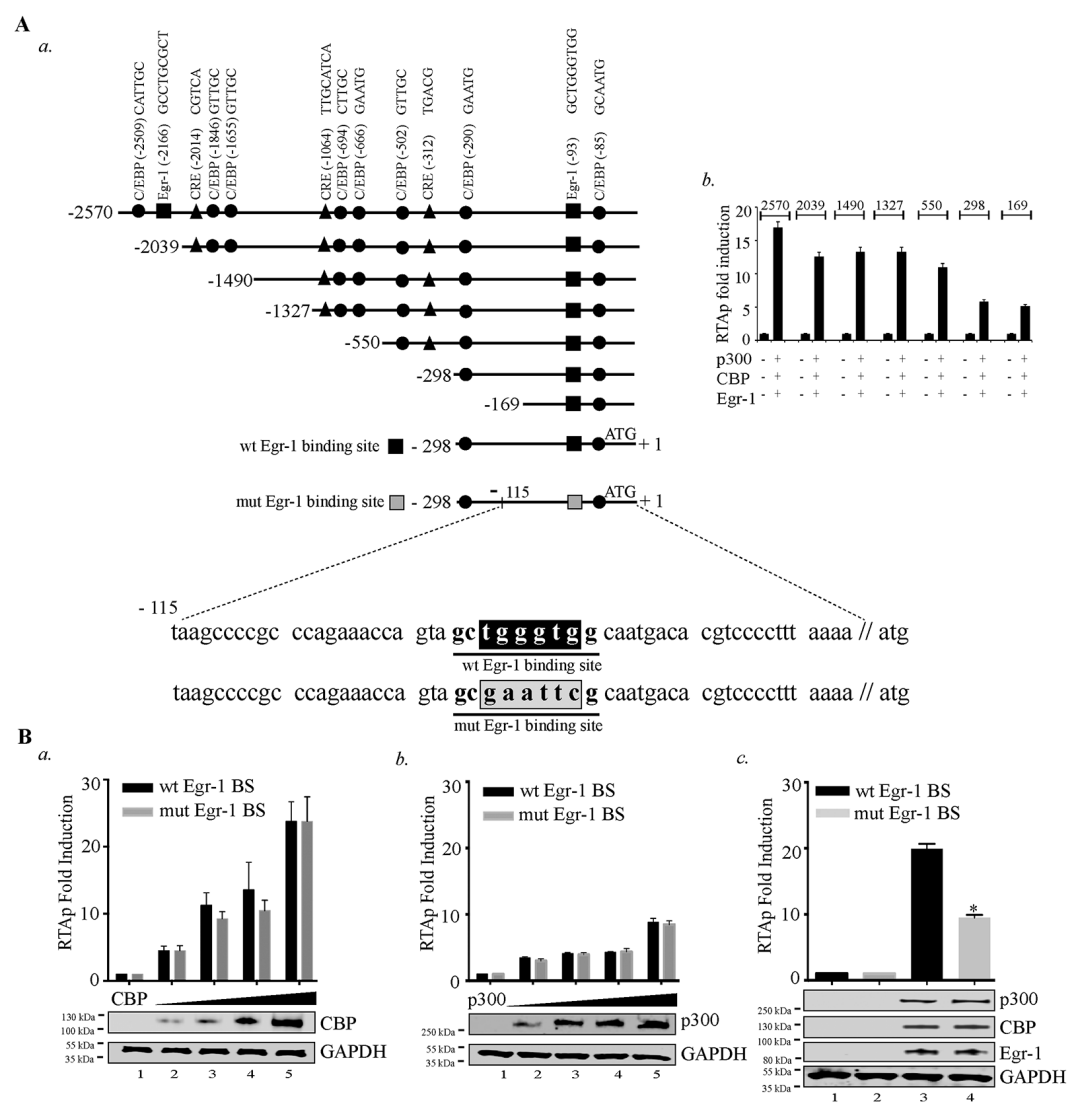
Disruption of Egr-1 binding site at RTA promoter reduces CBP/p300 mediated transcriptional activity **(A)** a. Schematic shows the deletion mutants of RTA promoter and the location of Egr-1 and CBP (CREB-binding protein) binding sites such as: C/EBPs - CCAAT-enhancer-binding proteins; CRE - cAMP response elements. b. A total of 2 × 10^6^ 293T cells were cotransfected with the combination of Egr-1 (0.5µg), CBP (0.5µg), p300 (0.5µg) and RTAp-2570 (0.1µg)/RTAp-2039 (0.1µg)/RTAp-1490 (0.1µg)/RTAp-1327 (0.1µg)/RTAp-500 (0.1µg)/RTAp-298 (0.1µg)/RTAp-169 (0.1µg). Lucifearse assay was performed 48h post-transfection and the relative luciferase units are presented as RTAp fold induction. **(B)** Luciferase reporter assay was performed to compare the RTA promoter (RTAp) activity with either wild type Egr-1 binding site (wt Egr-1 BS) or mutated Egr-1 binding site (mut Egr-1 BS). a. RTA promoter (wt Egr-1 BS/mut Egr-1 BS) activity was compared in the presence of increasing concentration of CBP (0.0, 0.2, 0.4, 0.6, 0.8 μg): Lanes 1-5, b. RTA promoter (wt Egr-1 BS / mut Egr-1 BS) activity was compared in the presence of increasing concentration of p300 (0.0, 0.2, 0.4, 0.6, 0.8 μg): Lanes 1-5, c. RTA promoter (with Egr-1/ Egr-1 mutated site) activity was determined in the presence of CBP, p300 and Egr-1. 293T cells were transfected with: lane 1- RTA promoter (with Egr-1 binding site) 0.1μg; lane 2- RTA promoter (mutated Egr-1 binding site) 0.1μg; lane 3- RTA promoter (with Egr-1 binding site) 0.1μg, CBP (0.5μg), p300 (0.5μg) and Egr-1 (0.5μg); lane 4- RTA promoter (mutated Egr-1 binding site) 0.1μg, CBP (0.5μg), p300 (0.5μg) and Egr-1 (0.5μg). Luciferase assay was performed 48h post-transfection and the relative luciferase units are presented as RTAp fold induction.

**Figure 5:**
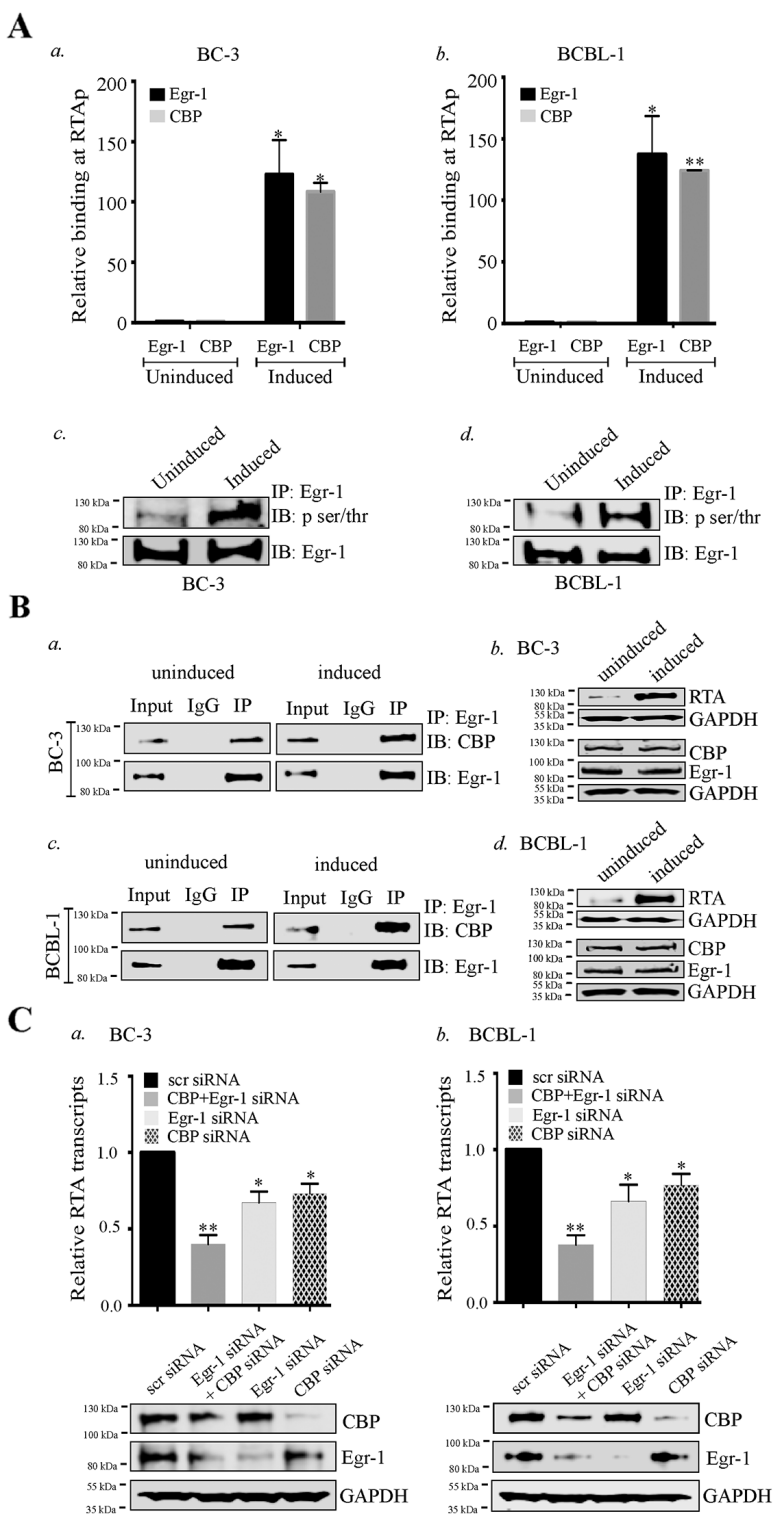
Egr-1 and CBP binds at RTA promoter (RTAp) and regulates its transcription during viral reactivation **(A)** 10 ×10^6^ BC-3 and 10 ×10^6^ BCBL-1 cells were cultured and induced with TPA for 24h. The cells were harvested and processed for Chromatin Immunoprecipitation assay. a. The relative binding of Egr-1 and CBP at RTA promoter was compared between uninduced and induced BC-3 cells. b. The relative binding of Egr-1 and CBP at RTA promoter was compared between uninduced and induced BCBL-1 cells. ^*^P < 0.05, ^**^P < 0.01. c. 10 ×10^6^ BC-3 cells were induced with TPA for 24h. The cells were harvested and processed for the detection of Egr-1 phosphorylation through immunoprecipitation and immunoblotting. d. 10 ×10^6^ BCBL-1 cells were induced with TPA for 24h. The cells were harvested and processed for the detection of Egr-1 phosphorylation through immunoprecipitation and immunoblotting. **(B)** The interaction of Egr-1 and CBP was compared between uninduced and induced (BC-3 and BCBL-1) cells. The cells were induced for 24h, harvested and processed for Egr-1 immunoprecipitation. Egr-1 and CBP were immunoprecipitated through Egr-1 antibody, as detected through immunoblotting. a. uninduced and induced BC-3. b. The respective RTA, Egr-1 and CBP expression levels are shown in uninduced and induced BC-3.Egr-1 and CBP were immunoprecipitated through Egr-1 antibody, as detected through immunoblotting, as detected through immunoblotting in c. uninduced and induced BCBL-1. d. The respective RTA, Egr-1 and CBP expression levels are shown in uninduced and induced BCBL-1. **(C)** a. BC-3, b. BCBL-1 cells were transfected with either scr/Egr-1/CBP/Egr-1 and CBP for 36h and induced with TPA for 24h. The cells were harvested and processed for the analysis of RTA transcripts levels through qPCR. The respective Egr-1, CBP and GAPDH expression are shown in each of the experimental sets. ^*^P < 0.05, ^**^ *P* < 0.01.

After verifying the cooperative effects of Egr-1, CBP/p300 in augmenting RTA promoter activity, we wanted to gain insight into the mechanism of RTA transcription by identifying the specific region of RTA promoter important for this effect. Accordingly, we used different truncation mutants of RTA promoter in reporter assays for cells expressing Egr-1, CBP and p300 (Figure 4A, *a*). We co-expressed these deletion mutants (2570, 2039, 1490, 1327, 550, 298 and 169bp) of RTA promoter in combination with Egr-1, CBP and p300 (Figure 4A, *b*). The results showed a reduction in promoter activity in 2039 truncation mutant from 2570 region. The deleted region contains an Egr-1 (distal) and C/EBP binding sites, which might have contributed to the reduction in the promoter activity. The next reduction in promoter activity was noted from 1327 to 550 truncations. Sequence analysis of this region confirmed the presence of C/EBPs (CCAAT-enhancer-binding proteins) [64] and CREs (cAMP response elements) [65] sites. Therefore, deleting that region negatively impacted the promoter activity. Similarly, truncation from 550 to 298 removed CRE and C/EBP binding sites, which may have contributed to a significant reduction in the promoter activity. The RTA promoter activity of 169 promoter region clearly indicated the presence of Egr-1 binding site within that region. (Figure 4A, *b*). Though, it was clear that Egr-1, CBP/p300 were capable of augmenting 169 bp RTA promoter, we wanted to confirm the significance of Egr-1 binding site on CBP/p300 mediated RTA promoter upregulation. To this end, we used the DNA binding mutant of Egr-1 at RTA promoter to confirm the direct role of Egr-1 in mediating RTA promoter activity. Additionally, we titrated CBP and p300 separately in the absence of Egr-1 expression (Figure 4B, *a* for CBP and *b* for p300) to analyze the effect of CBP and p300 on mutated Egr-1 site on RTA promoter. We found that both CBP and p300 could induce RTA promoter activity independently in a dose dependent manner. This dose responsive effect was unaltered in promoter with mutated Egr-1 site (Figure 4B, *a* for CBP and *b* for p300). This confirmed the importance of C/EBP binding site within the 298-promoter region. Importantly, mutation of Egr-1 binding site led to a suppression in the RTA promoter activity in the presence of Egr-1, CBP and p300 co-expression (Figure 4B, *c*). These results clearly validated the role of Egr-1 with respect to its specific binding at the RTA promoter. The current observation demonstrated various possibilities, which indicated that CBP/p300 could upregulate RTA promoter activity but the interplay of Egr-1 and CBP is essential in the enhancement of RTA promoter activity.

### The binding of Egr-1 and CBP at RTA promoter is enhanced during viral reactivation

Though the results from the reporter assays depicted the cooperative effect of Egr-1, CBP/p300 in regulating RTA promoter activity, the direct evidence pertaining to the binding of Egr-1 and CBP during the viral reactivation was still lacking. Therefore, we conducted ChIP assay during lytic induction to compare the binding efficiencies of Egr-1 and CBP at RTA promoter (Figure 5A, *a* for BC-3; *b* for BCBL-1). The assay revealed that the binding of Egr-1 and CBP at RTA promoter was significantly enhanced during the viral reactivation. The binding of p300 was also detected at RTA promoter during viral reactivation (data not shown). Here, we focused our study on CBP binding because the reporter assay showed that CBP predominantly augments RTA promoter activity as compared to p300, when co-expressed with Egr-1. While comparing the relative binding of Egr-1 and CBP, we found that the recruitment of CBP was lower than Egr-1 at RTA promoter in the reactivated samples (BC-3 and BCBL-1 cells). However, comparative binding of both Egr-1 and CBP at RTA promoter was higher in the induced cells than the uninduced cells (Figure 5A, *a* for BC-3, *b* for BCBL-1). As an additional evidence of Egr-1 and CBP binding at RTA promoter, our ChIP data supported the results from reporter assay depicting that Egr-1 and CBP are predominantly recruited at RTA promoter to regulate transcription.

We also analyzed the phosphorylation status of Egr-1 in BC-3 and BCBL-1 cells. The comparison was made between the phosphorylated form of Egr-1 from uninduced and induced cells (Figure 5A, *c* for BC-3, *d* for BCBL-1). Interestingly, the level of phosphorylated Egr-1 was higher in induced cells as compared to the respective uninduced cells with total Egr-1 showing similar expression (Figure 5A, *c* for BC-3, *d* for BCBL-1, IB: Egr-1 panel).

Since Egr-1 was shown to interact with CBP [24, 63, 66], so we wanted to determine whether these two proteins bind in KSHV infected cells undergoing lytic reactivation. The immunoprecipitation of Egr-1 co-precipitated CBP (Figure 5B, *a* for BC-3, *c* for BCBL-1) as well as p300 (data not shown) from the KSHV infected cells, which is consistent with the previous reports of Egr-1 binding to CBP and p300. Surprisingly, the comparative binding of CBP with Egr-1 was enhanced in cells undergoing lytic reactivation evidenced by a higher relative intensity of CBP in IP lanes of induced cells as compared to the uninduced cells (Figure 5B, *a* for BC-3, *c* for BCBL-1). We confirmed that the higher binding of CBP to Egr-1 was not due to a higher level of CBP in induced cells since the levels of CBP and Egr-1 were comparable between these two sets (Figure 5B, *b* for BC-3 and *d* for BCBL-1). These results indicated the possibility that Egr-1 phosphorylation could be responsible for its enhanced binding with CBP during viral reactivation. Taken together, these observations clearly supported the fact that RTA transcription is regulated by an enhanced association and recruitment of Egr-1 and CBP at RTA promoter.

Next, we wanted to verify whether Egr-1 and CBP were acting cooperatively in the regulation of RTA transcription. We transfected BC-3 and BCBL-1 cells with siRNA to deplete Egr-1 and CBP together or Egr-1 and CBP individually, 24h prior to the induction for lytic reactivation. These cells were induced for 24h before harvesting them for the detection of RTA transcripts. Relative copies of RTA transcripts showed a significant reduction in the cells depleted for Egr-1 or CBP (Figure 5C, *a* for BC-3, *b* for BCBL-1). Importantly, the cells depleted for both Egr-1 and CBP showed a much higher reduction in RTA transcripts as compared to the individually depleted cells (Figure 5C, *a* for BC-3, *b* for BCBL-1), hence confirming that Egr-1 and CBP act cooperatively to regulate RTA transcription during lytic induction.

### Egr-1 phosphorylation is upregulated during KSHV *de novo* infection

**Figure 6:**
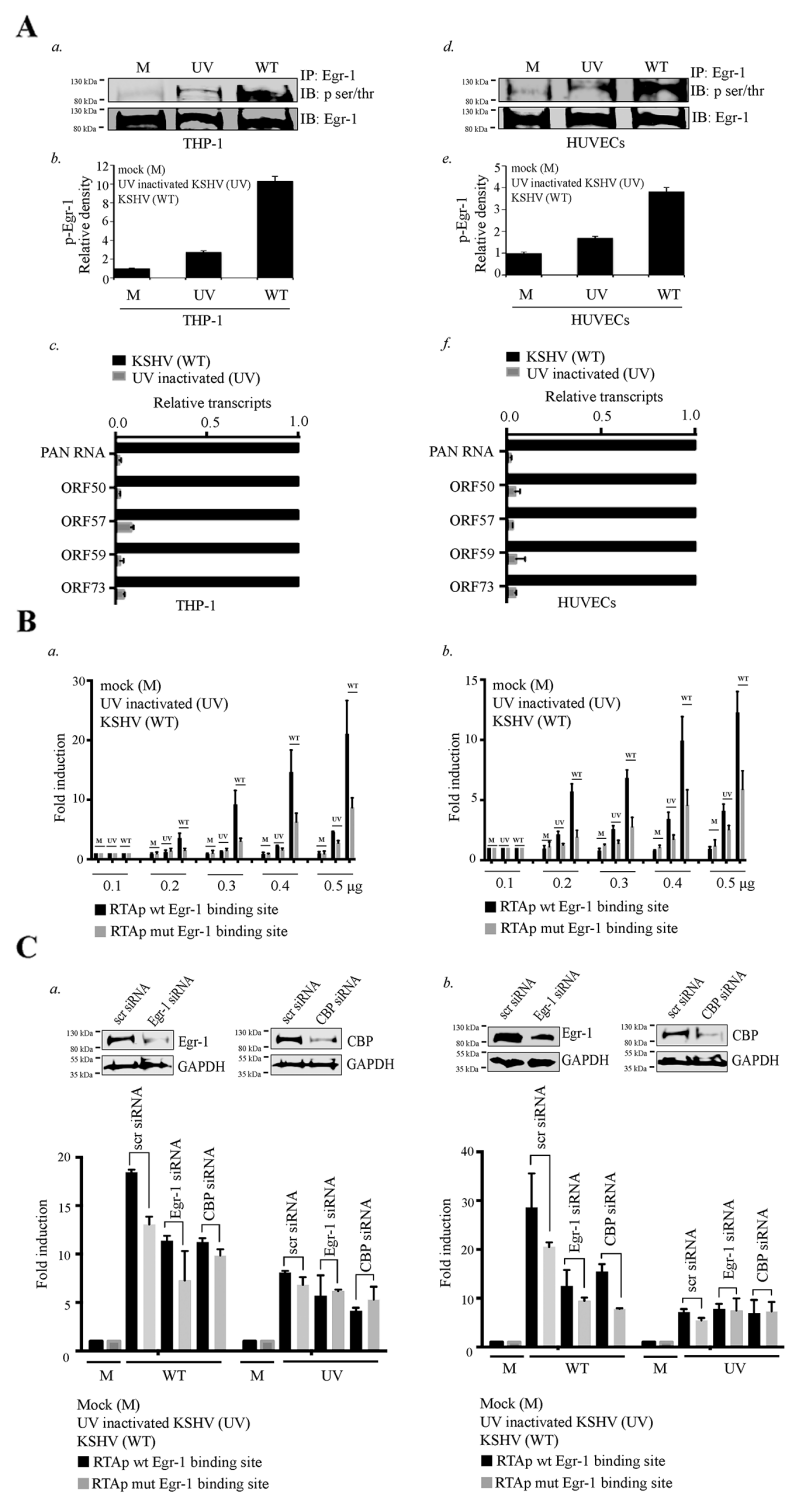
Egr-1 gets phosphorylated during primary infection. **(A)** 2×10^6^ THP-1 cells were infected with either live or UV inactivated KSHV at 10 m.o.i for 12h. a. The Egr-1 phosphorylation levels were compared between the target cells infected with either UV inactivated (UV) or live KSHV (WT). b. Relative density of p-Egr-1 levels from the THP-1 infected with (UV inactivated/live virus). c. Relative KSHV transcripts PAN RNA, ORF50, ORF57, ORF59 and ORF73 were compared after 12h of infection in THP-1. 2×10^6^ HUVECs were infected with either live or UV inactivated KSHV at 10 m.o.i for 12h. d. The Egr-1 phosphorylation levels were compared between the target cells infected with either UV inactivated or live KSHV. e. Relative density of p-Egr-1 levels from the HUVECs infected with (UV inactivated/ live virus). f. Relative KSHV transcripts PAN RNA, ORF50, ORF57, ORF59 and ORF73 were compared after 12h of infection in HUVECs. **(B)** a. 2×10^6^ THP-1 cells were transfected with the combination of either RTA promoter (with Egr-1 binding site) or RTA promoter (mutated Egr-1 binding site). The concentration of plasmids transfected were 0.1μg, 0.2μg, 0.3μg, 0.4μg and 0.5μg respectively. After transfection of RTA promoter plasmids, the target cells were infected with KSHV/UV treated KSHV at 10 m.o.i for 6h. The target cells were harvested and processed for luciferase assay. b. 2×10^6^ HUVECs were transfected with the combination of either RTA promoter (with Egr-1 binding site) or RTA promoter (mutated Egr-1 binding site). The concentration of plasmids transfected were 0.1μg, 0.2μg, 0.3μg, 0.4μg and 0.5μg respectively. After transfection of RTA promoter plasmids, the target cells were infected with KSHV/UV treated KSHV at 10 m.o.i for 6h. The target cells were harvested and processed for luciferase assay. **(C)** a. 2×10^6^ THP-1 cells were co-transfected with the combination of either scr/Egr-1/CBP siRNA (100 pM) and RTA promoter (0.1μg) (with Egr-1 binding site)/RTA promoter (mutated Egr-1 binding site). The expression levels of Egr-1/CBP with their respective GAPDH levels are shown in scr/Egr-1/CBP siRNA transfected THP-1. The target cells were infected with KSHV/UV treated KSHV at 10 m.o.i for 6h prior to harvesting. The cells were processed for luciferase assay. b. 2×10^6^ HUVECs were co-transfected with the combination of either scr/Egr-1/CBP siRNA (100 pM) and RTA promoter (0.1μg) (with Egr-1 binding site)/RTA promoter (mutated Egr-1 binding site). The expression levels of Egr-1/CBP with their respective GAPDH levels are shown in scr/Egr-1/CBP siRNA transfected HUVECs. The target cells were infected with KSHV/UV treated KSHV at 10 m.o.i for 6h prior to harvesting and processed for luciferase assay.

Since the expression and phosphorylation of Egr-1 is one of the key factors in regulating viral reactivation (Figure 1 and Figure 2), we wanted to determine whether Egr-1 gets phosphorylated during the initial burst of lytic cycle during *de novo* infection. To this end, we used a monocytic cells, THP-1 and endothelial cells, HUVECs for *de novo* infection as these cells represent a model for studying KSHV primary infection [67–70]. The infection of HUVECs and THP-1 with KSHV showed augmented Egr-1 phosphorylation when compared with the respective mock infected (M) cells (Figure 6A, *a* for THP-1 and *d* for HUVECs). Next, we verified whether active infection was responsible for Egr-1 phosphorylation by infecting them with an UV inactivated virus (UV), which showed a significantly reduced level of Egr-1 as compared to the cells infected with the live virus (Figure 6A, *a* for THP-1 and *d* for HUVECs). The densitometric analyses of the phosphorylated Egr-1 are shown in Figure 6A (*b* for THP-1 and *e* for HUVECs). Thus, active viral replication primarily contributes to Egr-1 phosphorylation apart from viral membrane attachment. We have also analyzed the active replication of KSHV via detection of viral transcripts from the infected cells at 12 hpi through a real-time PCR assay, which showed abundant viral transcripts in wild type (WT) virus infected cells as compared to the UV treated ones (Figure 6A, *c* for THP-1 and *f* for HUVECs). The results indicate an active replication following KSHV infection as a contributor of Egr-1 phosphorylation.

### RTA promoter with mutated Egr-1 site shows reduced activity during KSHV infection

After analyzing the status of Egr-1 phosphorylation during KSHV *de novo* infection, we wanted to determine the effect of Egr-1 mutation on RTA promoter activity during KSHV infection. The target cells transfected with RTA promoter containing intact or mutated Egr-1 binding site were infected with KSHV followed by analyzing the relative promoter activation. Comparative analysis revealed that RTA promoter with mutated Egr-1 site had reduced promoter activity at all the tested concentrations of promoter plasmid (Figure 6B, *a* for THP-1 and *b* for HUVECs). The infection was also conducted using UV inactivated virus and we found highest promoter augmentation with wild type virus. Our results also revealed that the UV inactivated virus was capable of enhancing the RTA promoter activity although at lower levels as compared to the live KSHV. This could possibly be due to the fact that Egr-1 becomes phosphorylated (through membrane fusion and viral replication) during primary *de novo* infection. Next, we determined the role of Egr-1 binding sites in RTA promoter activity during *de novo* KSHV infection. The above experiment was also conducted with Egr-1 mutated RTA promoter transfected cells. Relative fold induction showed a reduced promoter activity in cells transfected with Egr-1 mutated RTA promoter during KSHV infection. Altogether, these results confirmed that Egr-1 site of RTA promoter is essential for KSHV mediated RTA promoter activation during *de novo* infection.

### Egr-1 and CBP are involved in the regulation of RTA promoter activity during KSHV infection

We extended our study to analyze whether Egr-1 and CBP act cooperatively during the onset of KSHV infection. In order to achieve efficient transfection of siRNA in THP-1 and HUVECs, we used nucleofection [71]. The siRNA to deplete Egr-1 and CBP were transfected separately in the respective target cells (THP-1 and HUVECs). The efficiency of gene silencing was determined for CBP and Egr-1 through western blotting (Figure 6C, *a* for THP-1 and *b* for HUVECs) after 48h of transfection. The target cells were co-transfected with RTA promoter (either with Egr-1 or mutated Egr-1 site) plasmids along with specific siRNA. The siRNA transfected cells were infected with KSHV for 6 h and proceeded to luciferase assay. The results revealed that the target cells, transfected with control siRNA (scr siRNA) had an upregulation in the RTA promoter activity. We found a suppression in RTA promoter activity in the target cells with depleted Egr-1 levels after infection. Surprisingly, there was also a reduction in RTA promoter activity in the infected target cells with depleted CBP levels. Hence, it was clear from these experiments that both Egr-1 and CBP played significant roles in driving the RTA transcription during *de novo* KSHV infection. Parallely, we determined the levels of RTA promoter (mutated Egr-1 site) activation in the target cells (transfected with either depleted Egr-1 or CBP) after KSHV infection (Figure 6C, *a* for THP-1 and Figure 6C, *b* for HUVECs). Collectively, these data revealed that RTA promoter with Egr-1 mutated site had suppressed promoter activity (in the absence of either Egr-1 or CBP) when compared with the target cells co-transfected with scr siRNA and RTA promoter (with Egr-1 binding site) during *de novo* KSHV infection.

We also used UV treated virus to infect the target cells transfected with scr/Egr-1/CBP siRNA for assaying the RTA promoter activity. (Figure 6C, *a* for THP-1 and Figure 6C, *b* for HUVECs). The results showed no significant difference among the experimental sets. The fold induction in RTA promoter activity in the target cells co-transfected with a combination of either scr/Egr-1/CBP siRNA and RTA promoter (Egr-1 wt or mutated) was not significantly changed. (Figure 6C, *a* for THP-1 and Figure 6C, *b* for HUVECs). Hence, these observations indicated that Egr-1 and CBP could be cooperatively acting during KSHV *de novo* infection.

### Egr-1 expression regulates RTA transcription during primary *de novo* infection

**Figure 7:**
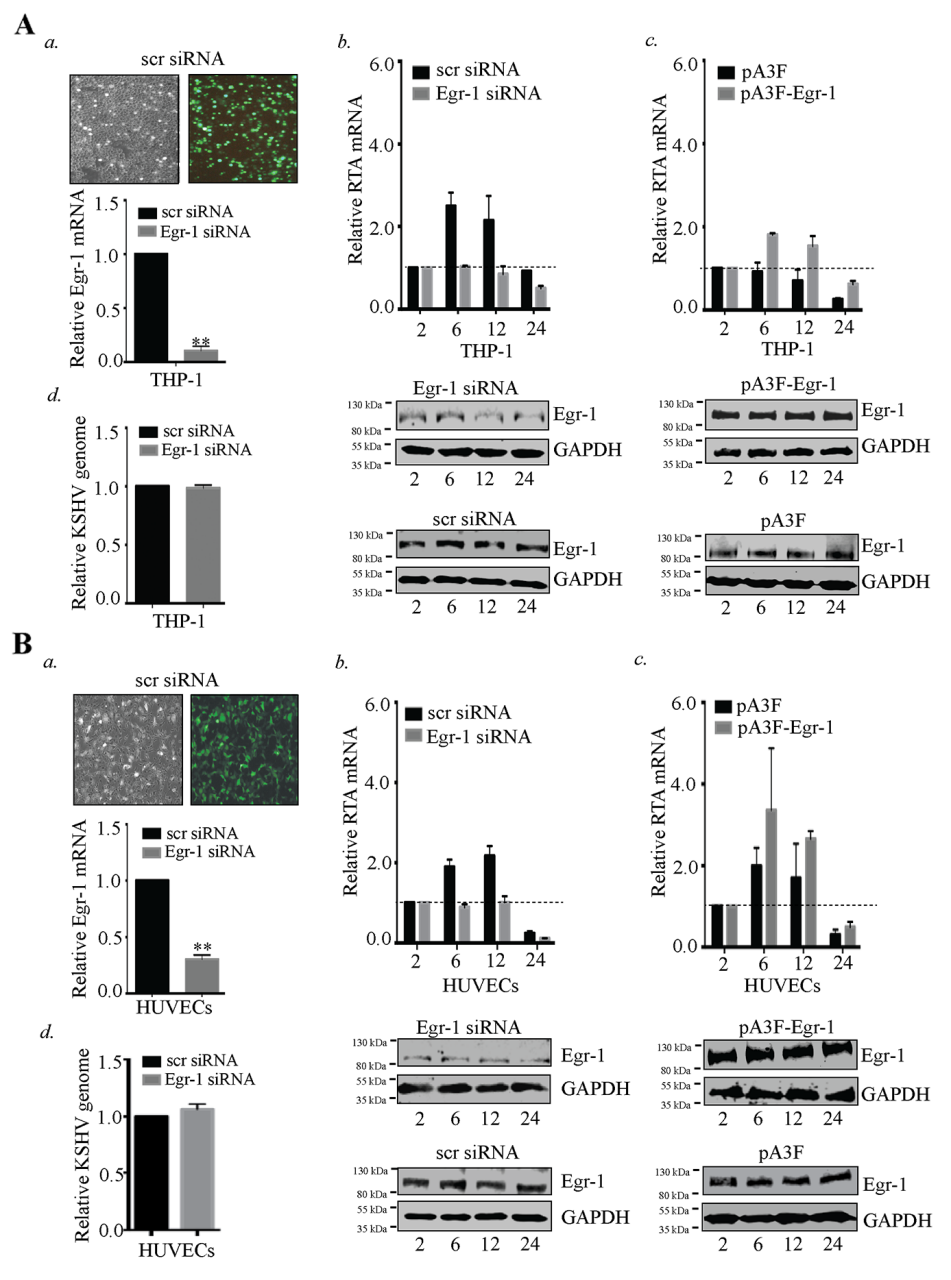
Egr-1 controls RTA transcription during primary infection. **(A)** 2×10^6^ THP-1 cells transfected with Egr-1 siRNA (100 pM). The target cells were infected with KSHV at 10 m.o.i for 2, 6, 12 and 24h. The cells were harvested at indicated time points and processed for the detection of RTA transcripts. a. Transfection efficiency of siRNA and the reduction of Egr-1 levels (due to Egr-1 siRNA transfection) are shown for THP-1. ^**^ *P* < 0.01. b. RTA transcripts levels were reduced in Egr-1 depleted THP-1 detected at 2, 6, 12 and 24h post-infection. c. 2×10^6^ THP-1 were transfected either with pA3F-Egr-1 (5µg) or control vector, pA3F (5µg) for 48h. The target cells were infected with KSHV at 10 m.o.i. Cells were harvested at indicated time points and processed for RTA transcripts analysis. Egr-1 expression level was compared between pA3F-Egr-1 transfected cells and control vector transfected cells. d. Silencing of Egr-1 does not alter KSHV entry in THP-1. KSHVDNA was extracted 2h post-infection from the THP-1 (scr siRNA and Egr-1 siRNA transfected cells). The extracted DNA was analyzed for the target gene (ORF58) amplification through qPCR. The relative KSHV genome copies were calculated and compared between KSHV infected cells (transfected with scr/Egr-1 siRNA). **(B)** 2×10^6^ HUVECs transfected with Egr-1 siRNA (100 pM). The target cells were infected with KSHV at 10 m.o.i for 2, 6, 12 and 24h. The cells were harvested at indicated time points and processed for the detection of RTA transcripts. a. Transfection efficiency of siRNA and the reduction of Egr-1 levels (due to Egr-1 siRNA transfection) are shown for HUVECs. ^**^P < 0.01. b. RTA transcript levels were reduced in Egr-1 depleted HUVECs detected at 2, 6, 12 and 24h post-infection. The expression level of Egr-1 was reduced in Egr-1 siRNA (Egr-1 siRNA) transfected cells as compared to control (scr siRNA) cells. c. 2×10^6^ HUVECs were transfected either with pA3F-Egr-1 (5µg) or control vector, pA3F (5µg) for 48h. The target cells were infected with KSHV at 10 m.o.i. Cells were harvested at indicated time points and processed for RTA transcripts analysis. Egr-1 expression level was compared between pA3F-Egr-1 transfected cells and control vector transfected cells. d. Silencing of Egr-1 does not alter KSHV entry in HUVECs. KSHVDNA was extracted 2h post-infection from the infected HUVECs (scr siRNA and Egr-1 siRNA transfected cells). The extracted DNA was analyzed for the target gene (ORF58) amplification through qPCR. The relative KSHV genome copies were calculated and compared between KSHV infected cells transfected with scr/Egr-1 siRNA.

To study the direct effects of Egr-1 expression during KSHV infection, THP-1 and HUVECs cells were depleted of Egr-1 by transfecting specific siRNA for Egr-1 or scrambled siRNA as a control. The depletion of Egr-1 in the target cells was confirmed through amplification of Egr-1 specific transcripts by RT-PCR (Figure 7A, *a* for THP-1 and Figure 7B, *a* for HUVECs).

The expression of RTA, which is regulated through Egr-1, was analyzed at different time intervals (2, 6, 12 and 24h) post-infection. The data showed a significant reduction in RTA transcription after KSHV infection in the Egr-1 depleted THP-1 cells (Figure 7A, *b*). Similarly, HUVECs depleted of Egr-1 showed reduced RTA transcripts as compared to the control siRNA (scr siRNA) transfected cell (Figure 7B, *b*). The respective levels of Egr-1 expression in siRNA-transfected cells following KSHV infection are shown in Figure 7A, *b* for THP-1 and Figure 7B, *b* for HUVECs. We further confirmed the significance of Egr-1 expression on RTA transcription during KSHV primary infection by infecting THP-1 and HUVECs overexpressing Egr-1. Interestingly, the levels of RTA transcripts in Egr-1 overexpressing cells were enhanced as compared to the control cells (Figure 7A, *c* for THP-1 and Figure 7B, *c* for HUVECs) after KSHV infection. The expression levels of Egr-1 and respective GAPDH at different time points (2, 6, 12 and 24h) post-infection are shown in THP-1 and HUVECs transfected with either pA3F-Egr-1 or pA3F (empty vector) (Figure 7A, *c* for THP-1 and 7B, *c* for HUVECs). Therefore, Egr-1 actively regulates RTA transcription during primary *de novo* infection.

Our results demonstrated that depletion of Egr-1 suppresses RTA transcription. Also, it is known that Egr-1 expression gets regulated through various signaling cascades such as c-Abl [72], PI3K [73], that are involved in KSHV entry [74]. Therefore, we were keen to know whether depletion of Egr-1 could affect KSHV entry into the target cells. To ascertain the role of Egr-1 in modulating KSHV entry, we transfected the target cells with Egr-1 siRNA (24h prior to KSHV infection) (Figure 7A, *d* for THP-1 and Figure 7B, *d* for HUVECs). After 2h post infection, the cells were harvested for viral genome extraction. Internalized viral genome was quantified by amplifying ORF58 gene and the relative virion copies in Egr-1 siRNA transfected cells were compared with control siRNA transfected cells. We did not find any significant change in the relative KSHV genome copies between the Egr-1 siRNA and scr siRNA transfected cells following infection. Thus, it was clear that Egr-1 depletion did not affect viral entry into the target cells during *de novo* infection (Figure 7A, *d* for THP-1 and Figure 7B, *d* for HUVECs).

## DISCUSSION

The current study focuses on the mechanisms underlying Egr-1 mediated lytic activation in KSHV life cycle. Egr-1 is involved in various cellular processes and the studies from herpesviruses show that radiosensitive Egr-1 promoter activation could induce the expression of thymidine kinase gene in HSV-1 [75, 76]. Egr-1 is also capable of suppressing the transcription of HSV-1 latency-associated transcript [43] by binding to TATA binding protein (TBP) at the promoter region [47], which indicates the functional role of Egr-1 in augmenting viral reactivation. Additional reports have documented the role of Egr-1 in maintaining HSV-1 latency by suppressing the expression of immediate early gene [77]. This effect of Egr-1 is achieved through the binding of Nab2 at the promoter region instead of Sp1, leading to gene silencing. Egr-1 is also known to enhance the lethality of HSV-1 by augmenting viral replication [78]. Overexpression of Egr-1 causes hepatic stromal keratitis (HSK), which gets significantly suppressed in the absence of Egr-1 due to a reduction in viral replication [79]. Also, Egr-1 expression gets enhanced during the lytic replication of HSV-1 due to the recruitment of CREB and NFκB at Egr-1 promoter [40]. HSV-1 replication correlates with Egr-1 expression and its occupancy at viral regulatory sequences [80].

A significant role of Egr-1 has been demonstrated in the life cycle of other viruses including EBV where it induces the expression of immediate early gene, BRLF1 [81]. The rapid expression of Egr-1 in regulating unrestricted viral gene expression through occupancy at the consensus sequences after EBV infection has also been demonstrated [82]. Egr-1 expression is upregulated through EBV lytic transactivator, Zta, which supports lytic replication [45]. Zta modulates promoter activity through its interaction with methylated Zta-response elements (ZRE) at Egr-1 promoter [83]. It has also been reported that the latent membrane protein 1 (LMP1) positively regulates Egr-1 through the involvement of NFκB in order to mediate LMP1 dependent cancer cell survival [84]. The blockage of Hodgkin’s lymphoma cells from entering into the EBV lytic cycle also occurs through the suppression of Egr-1 [85]. Taken together, these studies demonstrate the functions of Egr-1 in regulating lytic as well as latent phases of the viral life cycle.

**Figure 8:**
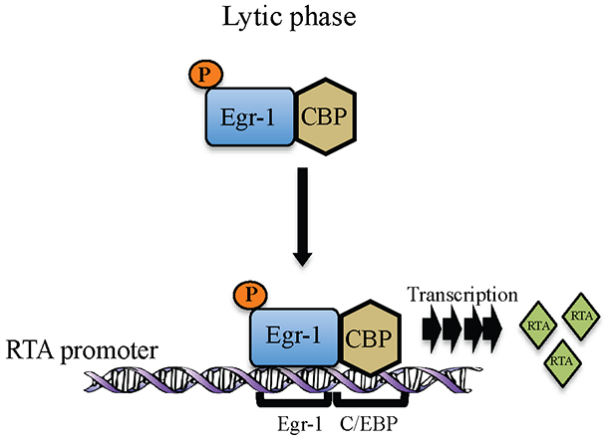
Schematic showing Egr-1 phosphorylation and formation of Egr-1 and CBP complex at RTA promoter during lytic reactivation for the transcription of RTA

In KSHV, Egr-1 has been shown to mediate RTA promoter activity [21]. Data presented here adds to our understanding of how Egr-1 modulates promoter activity by recruiting other cellular factors. The roles of CBP and p300 are very well defined in KSHV biology and these transcription factors are shown to interact with RTA independently to modulate transcriptional activity [49]. Also, the interaction of Egr-1 with CBP and p300 has been shown *in vivo* [24]. The roles of transcriptional coactivators reported here add significant information on the KSHV life cycle since they could modulate Egr-1 mediated RTA transcription. The interaction of EGR-1 with the transcriptional coactivators, CBP/p300 resulted in the enhancement of the transcriptional activity. Our reporter assay revealed that Egr-1 could highly upregulate RTA promoter activity in the presence of transcriptional coactivators, CBP and p300. Additionally, the ChIP analysis demonstrated that Egr-1 and CBP were recruited at RTA promoter during lytic induction. Though Egr-1 and CBP binds in non-induced KSHV infected cells but the relative binding between Egr-1 and CBP gets enhanced during induction (Figure 5). The enhancement in Egr-1 phosphorylation as well as its increased binding with CBP could be the consequences of viral reactivation. Hence, these observations indicate a correlation between Egr-1 phosphorylation and its interaction with CBP. Our results also showed that depletion of either Egr-1 or CBP can downregulate RTA transcription (upto ∼ 30% in Egr-1 depleted cells and ∼ 20% in CBP depleted cells); whereas depletion of both Egr-1 and CBP can reduce the RTA transcript levels upto ∼ 55% in both BC-3 and BCBL-1 cells after induction. Thus, these results strongly suggest a cooperative effect of Egr-1 and CBP at RTA promoter. The synergy of Egr-1 and CBP in regulating RTA transcription raises the possibility that Egr-1 gets phosphorylated during the lytic phase of KSHV, which enhances its interaction with CBP. The schematic of our working model of Egr-1 phosphorylation and it’s binding with CBP at RTA promoter during lytic reactivation is depicted in figure 8.

The data presented here supports earlier reports of Egr-1 phosphorylation and an enhancement in the DNA binding activity to control the transcription of target genes [53, 54]. We found that Egr-1 gets phosphorylated during lytic induction in BC-3 and BCBL-1 cells and Okadaic acid treatment can also induce Egr-1 phosphorylation. However, Okadaic acid in combination with p38MAP kinase inhibitor or Raf inhibitor (GW5074) leads to a reduction in Egr-1 phosphorylation. Therefore, our findings implicate the involvement of p38 and Raf kinases in the regulation of Egr-1 phosphorylation. Also, enhanced Egr-1 phosphorylation leads to higher copies of virion produced as compared to the ones with suppressed Egr-1 phosphorylation. Altogether, this study verifies the role of Egr-1 phosphorylation in KSHV virion production with the involvement of p38 MAP kinase and Raf pathway. Off note, it will be interesting to investigate various viral and cellular factors, which regulate Egr-1 phosphorylation.

Interestingly, Okadaic acid can also inhibit serine/threonine phosphatases 1 and 2A, activate protein kinase (PKC) and elevate the expression of hypoxia-inducible factor-1 (HIF-1), which may have a broader impact on cell signaling other than Egr-1 phosphorylation [86-88]. Additional studies have indicated the involvement of PKC and HIF-1 during KSHV reactivation [89, 90]. The role of Okadaic acid in Epstein-Barr virus early antigen (EBV-EA) expression has been documented earlier [91]. Although Okadaic acid is not a strong inducer of viral reactivation as compared to other chemical inducers such as 12-O-Tetradecanoylphorbol-13-acetate and sodium butyrate (NaB), [92] it can augment viral reactivation. Therefore, Okadaic acid could be used to decipher the involvement of various kinases involved in the regulation of Egr-1 phosphorylation in KSHV infected cells.

In the context of *de novo* KSHV infection, our analysis reveals that Egr-1 gets phosphorylated after infection of the target cells. Additionally, the comparison of Egr-1 phosphorylation between the target cells infected with either live or UV inactivated virus show highly enhanced Egr-1 phosphorylation in the cells infected with live virus. These observations clarify the fact that although membrane fusion can up-regulate Egr-1 phosphorylation, the replicating virus is mainly responsible for enhanced phosphorylation of Egr-1 during KSHV infection. We have studied the role of Egr-1 and CBP in regulating RTA promoter activity during primary infection. We and others have showed that Egr-1 and CBP bind at RTA promoter and augment its activity [21, 49]. Our data demonstrate that both Egr-1 and CBP are involved in regulating RTA promoter activity during KSHV primary infection. Also, the mutation of Egr-1 binding site of RTA promoter suppressed RTA promoter activity during primary infection. We also found that target cells transfected with Egr-1 or CBP siRNA showed reduced RTA promoter activity when compared with scr siRNA transfected cells. Taken together, our findings reveal that both Egr-1 and CBP are involved in the regulation of RTA promoter activity during *de novo* primary infection.

We determined that Egr-1 does not affect viral entry into the target cells. However, cellular kinases, p38 and ERK are known to be involved in viral entry and these kinases are also able to regulate Egr-1 activity [93]. Thus, the upstream effectors, which control Egr-1 phosphorylation are capable of regulating viral entry but not Egr-1 itself.

We also validated the correlation between Egr-1 expression and RTA transcription during primary infection by infecting the Egr-1 depleted cells with KSHV. The Egr-1 depleted cells infected with KSHV showed a significant reduction in RTA transcripts level. This observation was again confirmed by overexpressing Egr-1 in the target cells (THP-1 and HUVECs) before infecting with KSHV, which showed an enhanced RTA transcription. The present report indicate that Egr-1 phosphorylation could be a key factor for regulating KSHV lytic phase. The balance between phosphorylated and non-phosphorylated forms of Egr-1 might be an essential criterion for the maintenance of latent and lytic states of KSHV. One of the interesting facts displayed that the cells with depleted Egr-1 did not completely block the transcription of RTA. This basal level of RTA transcription in Egr-1 depleted cells indicates the role of RTA in its auto regulation along with various viral and cellular factors involved in driving RTA transcription besides Egr-1 [49, 94–97]. Taken together, the results suggest that Egr-1 could serve as a key switch protein of KSHV lytic reactivation. The current study also opens various aspects of future studies such as exploring the detailed mechanism of the formation of transcription complex (Egr-1/ CBP/ p300) at RTA promoter, characterizing the specificity of kinases involved in the modulation of Egr-1 activity and analyzing the function of Egr-1 in regulating different KSHV genes.

## MATERIALS AND METHODS

### Cell culture, plasmids and antibodies

BC-3 and BCBL-1 cells were cultured in RPMI1640 supplemented with 8% FBS. 293T cells were cultured in DMEM supplemented with 8% FBS. The THP-1 cells were maintained in RPMI1640 with 10% FBS. The HUVECs were cultured in endothelial growth medium (M200, ThermoFisher Scientific) with 10% FBS at 37°C with 5% CO2. The BJAB cells stably infected with recombinant KSHV.219 (referred to as Brk.219) were cultured in RPMI1640 supplemented with 10% FBS in the presence of 4.2 μg/ml puromycin as described elsewhere [98]. pA3F-Egr-1- Flag was constructed by sub-cloning Egr-1 from pMX-Egr-1 (Addgene) using primers S-Egr-1 (HindIII)-5′-AACTTAAGCTTCATGGCGGCGC-3′; AS-Egr-1 (EcoRI) 5′ CTGCAGAATTCCGCAAATTTCAA-3′. pCMV5-CBP-Flag (provided by David Lebrun, Division of Cancer Biology and Genetics, Cancer Research Institute, Queen’s University, Kingston, ON), pGL3-RTA promoter, pCMV-p300-myc (Addgene). RTA promoter deletion mutants; 2570, 2039, 1490, 1327, 550 were provided by Dr. Erle S. Robertson (University of Pennsylvania, USA), 298 and 298 (-93 TGGGTG) was a kind gift from Dr. Paul Lieberman (Wistar Institute, Philadelphia); and 169 was a kind gift from Dr. Adrian Whitehouse (University of Leeds, UK). The specific antibodies for Egr-1 and CBP were purchased from Santa Cruz Biotechnology. Anti-myc and anti-Flag antibodies were obtained commercially (ThermoFisher, Inc.) and Sigma Aldrich (Sigma Aldrich, Inc.), respectively.

### Luciferase reporter assay

Approximately 2 × 10^6^ 293T cells were grown overnight per well in a 6 well plate. The target cells were transiently transfected with specific plasmids using Metafectene (Biontex Laboratories) as described previously [99]. Twenty-four hours post-transfection, the medium was changed and incubated with fresh medium. The cells were harvested 48h post-transfection, lysed and used for the reporter assay as described previously [99]. Results presented were the average of triplicate experiments with the S.D. values shown as the error bars.

### Immunoprecipitation and immunoblotting

The target cells were harvested, washed with ice-cold phosphate-buffered saline (PBS), and lysed in 0.5 ml ice-cold radioimmunoprecipitation assay (RIPA) buffer (1% Nonidet P-40 [NP-40], 10 mM Tris [pH 7.5], 2 mM EDTA, 150 mM NaCl) supplemented with protease inhibitors (1 mM phenylmethylsulfonyl fluoride, 1µg/ml aprotinin, 1µg/ml pepstatin, 1µg/ml leupeptin). Cellular debris was removed by centrifugation at 13,000 × *g* for 10 min at 4°C, for immunoprecipitation, the lysate was precleared by 1h of rotation at 4°C with 30µl of protein A/G agarose beads. Approximately 100μl of the lysate was saved as an input control. The protein of interest was captured through incubation with 1µg of the appropriate antibody for overnight at 4°C rotator. The immune complexes were captured with a mixture of 30µl of protein A/G agarose beads at 4°C. The beads (containing bound proteins) were pelleted and washed three times with RIPA buffer. The immunoprecipitated proteins were further washed with RIPA buffer containing 300 mM NaCl to eliminate any contaminating proteins. For western blot analysis, the input lysates and immunoprecipitated complexes were boiled for 5 min in 1X Laemmli buffer, resolved through SDS-PAGE. The proteins were electroblotted to the nitrocellulose membrane, probed with appropriate antibodies, washed and then developed with infrared dye-tagged secondary antibodies using Odyssey Imager (LICOR).

### Chromatin immunoprecipitation assay

A total of 10 × 10^6^ BC-3 and 10 × 10^6^ BCBL-1 (uninduced/induced) cells were treated with a final concentration of 1% formaldehyde and cross-linked for 10 min at room temperature. Crosslinking was stopped by the addition of glycine at a final concentration of 0.125 M for 5 min. The cross-linked cells were washed in 1X PBS and counted so that approximately ∼ 8 × 10^6^ cells were used in each immunoprecipitation reactions as mentioned earlier [99]. Nuclei from the cells were isolated, lysed to collect chromatin and sheared to ∼300 bp. Lysates containing the chromatin was pre-cleared using 50µl of Protein A/G agarose beads in dilution buffer for 30 min at 4°C. The samples were centrifuged to recover the lysate by removing the beads. After aliquoting 100μl for the input controls, specific primary antibodies were added to the experimental samples and incubated overnight at 4°C with constant rotation. The DNA/protein complexes bound to the specific antibodies were immunoprecipitated using protein A/G beads with constant rotation for 2h at 4°C. The samples were washed using ChIP wash buffers followed by eluting and treating with proteinase K (1U) and incubating at 65°C overnight to reverse the crosslinks. The extracted DNA was purified using phenol/chloroform extraction and finally resuspended in 1X TE buffer. The extracted DNA was used for detecting the binding of Egr-1 and CBP at RTA promoter using specific primers spanning Egr-1 binding site through RT-PCR. The primers used were ORF50 forward, 5′-CTACCGGCGACTCATTAAG-3′, and ORF50 reverse, 5′-GTGGCTGCCTGGACAGTAT-3′ (125 bp product).

### RNA isolation, cDNA synthesis and RT-PCR

The total RNA was extracted using Illustra RNA spin Mini kit (GE Healthcare) following the manufacturer’s instructions and cDNA was synthesized using a High Capacity cDNA reverse transcription kit (Applied Biosystems). Reaction mixture for PCR consisted of (10μl of 2X PCR master mix (Applied Biosystems), 1μM primers (forward/reverse) and 2μl of cDNA. The synthesized cDNA was subjected to amplification on an ABI StepOne Plus real-time PCR machine (Applied Biosystems). The relative gene copy numbers/transcripts were calculated by the ΔΔ*CT* method. The reaction parameters for the detection of transcripts through Real time PCR (qPCR) was as follows: 95°C for 5 min, PCR cycling at 95°C for 10 s and 60°C for 30 s for 40 cycles. The gene expression data was normalized to the levels of housekeeping gene, GAPDH. The primers used for the detection of Egr-1 transcripts were Egr-1 S- 5′ CACCTGCATCTCACAGCCACT-3′; Egr-1 AS- 5′ GCCAACCCAAGCAGGAAGA-3′; RTA S-5′-CTACCGGCGACTCATTAAG-3′; AS-5′-GTGGCTGCCTGGACAGTAT-3′ and GAPDH were GAPDH S- 5′-CCCCTGGCCAAGGTCATCCA-3′ and AS- 5′-ACAGCCTTGGCAGCGCCAGT-3′.

### Viral genome extraction and quantification

The culture supernatant from 100 × 10^6^ BC-3/BCBL-1 cells was used for quantifying the virions produced after induction. Virions were concentrated through ultracentrifugation at 25000 rpm for 2h at 4°C. The concentrated virion pellet was resuspended in 1 ml of RPMI1640, containing 2% FBS and stored at −80°C. About 100μl of the resuspended virions were used for viral DNA extraction and quantification [18]. The KSHV ORF58 plasmid was used as a reference. We quantified virions using specific primers for ORF58 gene forward 5′-CTTAACTGTTTACTGCTGTTACCT-3′ and reverse 5′-AGACCTTCTTAAACAACAGAAGG-3′.

### siRNA transfection and gene silencing

A total of 2×10^6^ HUVECs/ 2×10^6^ THP-1 was plated per well in 6 well plates. The Egr-1 siRNA (100 pmol) and CBP siRNA (100 pmol) (Santa Cruz Biotechnology) were transfected using Amaxa Nucleofector Kit following specific programs U-001 for HUVECs, V-001 for THP-1 according to the manufacturer's instruction, T-001 for BC-3 and BCBL-1 as previously described [100]. For co-transfection of siRNAs, a total of 100 pmol (Egr-1) and 100 pm (CBP) were mixed together and transfected using the above-mentioned program for BC-3 and BCBL-1. The protocol for nucleofection was followed according to the instructions provided by manufacturer (Nucleofector Technologies, Lonza). The FITC conjugated scrambled siRNA (100 pmol) (Santa Cruz Biotechnology) was used as control for monitoring the efficiency of siRNA transfection. The medium was removed after 12h of siRNA transfection and replaced with fresh RPMI 1640 supplemented with 10% FBS for THP-1 and endothelial growth medium (M200, ThermoFisher Scientific) with 10% FBS for HUVECs. The efficiency of Egr-1/CBP silencing was checked after 48h of siRNA transfection through western blotting.

### Infection of HUVEC and THP-1 cells with KSHV

Briefly, THP-1 was infected with concentrated stock of KSHV following treatment with polybrene (5μg/ml). The cells were spun down for 5 min at 1200 rpm prior to infection. The culture supernatant was discarded and the cells were resuspended in 2ml of RPMI complete medium with virions. The mixture containing THP-1 was plated at density of 2×10^6^ cells per well of a 6 well plate. Rest of the protocol was followed as described earlier for THP-1 infection [68]. For HUVECs, the infection was carried out with KSHV in the presence of polybrene as described elsewhere [70]. The infection of HUVECs and THP-1 with KSHV at 10 m.o.i was carried out for 12h followed by RNA extraction and amplification of specific KSHV transcripts, PAN RNA, ORF50, ORF57, ORF59 and ORF73. The protocol and primers for the amplification KSHV transcripts were described elsewhere [18].

KSHV viral stocks were subjected to UV inactivation (1,200 μJ) in a UV Stratalinker 2400 (Stratagene). For viral entry experiments, THP-1 and HUVECs were infected with KSHV for 2h. The cells were harvested and proceeded for genome extraction as described earlier [18]. The target gene ORF58 was used as a reference and amplified to analyze the relative genome quantity.

### Cell viability analysis

The assay was performed as previously described [101]. Briefly, Cells were treated with specific inhibitors (Okadaic acid, p38 MAP Kinase inhibitor, GW5074) for 24h, washed twice with PBS, fixed with 10% formaldehyde for 10 min at room temperature. Fixed cells were stained with 0.1% crystal violet for 2 h at room temperature. Excess stain was washed with deionized water and the samples were allowed to dry at room temperature. The cells were counted using haemocytometer.

### Statistics

The data were statistically analyzed using GraphPad Prism software (GraphPad Software). *P* value < 0.05 (^*^) and *P* value < 0.01 (^**^) were considered statistically significant.

## References

[1] Soulier J, Grollet L, Oksenhendler E, Cacoub P, Cazals-Hatem D, Babinet P, d’Agay MF, Clauvel JP, Raphael M, Degos L (1995). Kaposi’s sarcoma-associated herpesvirus-like DNA sequences in multicentric Castleman’s disease. Blood.

[2] Cesarman E (2014). Gammaherpesviruses and lymphoproliferative disorders. Annu Rev Pathol.

[3] Cai Q, Verma SC, Lu J, Robertson ES (2010). Molecular biology of Kaposi’s sarcoma-associated herpesvirus and related oncogenesis. Adv Virus Res.

[4] Chang Y, Cesarman E, Pessin MS, Lee F, Culpepper J, Knowles DM, Moore PS (1994). Identification of herpesvirus-like DNA sequences in AIDS-associated Kaposi’s sarcoma. Science.

[5] Wang HW, Trotter MW, Lagos D, Bourboulia D, Henderson S, Makinen T, Elliman S, Flanagan AM, Alitalo K, Boshoff C (2004). Kaposi sarcoma herpesvirus-induced cellular reprogramming contributes to the lymphatic endothelial gene expression in Kaposi sarcoma. Nat Genet.

[6] Verma SC, Robertson ES (2003). Molecular biology and pathogenesis of Kaposi sarcoma-associated herpesvirus. FEMS Microbiol Lett.

[7] Coscoy L (2007). Immune evasion by Kaposi’s sarcoma-associated herpesvirus. Nat Rev Immunol.

[8] Lee HR, Brulois K, Wong L, Jung JU (2012). Modulation of Immune System by Kaposi’s Sarcoma-Associated Herpesvirus: Lessons from Viral Evasion Strategies. Front Microbiol.

[9] Guito J, Lukac DM (2012). KSHV Rta Promoter Specification and Viral Reactivation. Front Microbiol.

[10] Lukac DM, Yuan Y. (2007). Reactivation and lytic replication of KSHV. In: Arvin A, Campadelli-Fiume G, Mocarski E, Moore PS, Roizman B, Whitley R and Yamanishi K, eds. Human Herpesviruses: Biology, Therapy, and Immunoprophylaxis. (Cambridge University Press) 21348071

[11] Deng H, Liang Y, Sun R (2007). Regulation of KSHV lytic gene expression. Curr Top Microbiol Immunol.

[12] Purushothaman P, Dabral P, Gupta N, Sarkar R, Verma SC (2016). KSHV Genome Replication and Maintenance. Front Microbiol.

[13] Matsumura S, Fujita Y, Gomez E, Tanese N, Wilson AC (2005). Activation of the Kaposi’s sarcoma-associated herpesvirus major latency locus by the lytic switch protein RTA (ORF50). J Virol.

[14] Bowser BS, Morris S, Song MJ, Sun R, Damania B (2006). Characterization of Kaposi’s sarcoma-associated herpesvirus (KSHV) K1 promoter activation by Rta. Virology.

[15] Yang Z, Yan Z, Wood C (2008). Kaposi’s sarcoma-associated herpesvirus transactivator RTA promotes degradation of the repressors to regulate viral lytic replication. J Virol.

[16] Wang SE, Wu FY, Yu Y, Hayward GS (2003). CCAAT/enhancer-binding protein-alpha is induced during the early stages of Kaposi’s sarcoma-associated herpesvirus (KSHV) lytic cycle reactivation and together with the KSHV replication and transcription activator (RTA) cooperatively stimulates the viral RTA, MTA, and PAN promoters. J Virol.

[17] Chang PJ, Shedd D, Gradoville L, Cho MS, Chen LW, Chang J, Miller G (2002). Open reading frame 50 protein of Kaposi’s sarcoma-associated herpesvirus directly activates the viral PAN and K12 genes by binding to related response elements. J Virol.

[18] Purushothaman P, Thakker S, Verma SC (2015). Transcriptome analysis of Kaposi’s sarcoma-associated herpesvirus during de novo primary infection of human B and endothelial cells. J Virol.

[19] Krishnan HH, Naranatt PP, Smith MS, Zeng L, Bloomer C, Chandran B (2004). Concurrent expression of latent and a limited number of lytic genes with immune modulation and antiapoptotic function by Kaposi's sarcoma-associated herpesvirus early during infection of primary endothelial and fibroblast cells and subsequent decline of lytic gene expression. J Virol.

[20] Purushothaman P, Uppal T, Verma SC (2015). Molecular biology of KSHV lytic reactivation. Viruses.

[21] Dyson OF, Traylen CM, Akula SM (2010). Cell membrane-bound Kaposi’s sarcoma-associated herpesvirus-encoded glycoprotein B promotes virus latency by regulating expression of cellular Egr-1. J Biol Chem.

[22] Dyson OF, Walker LR, Whitehouse A, Cook PP, Akula SM (2012). Resveratrol inhibits KSHV reactivation by lowering the levels of cellular EGR-1. PLoS One.

[23] Sukhatme VP, Cao XM, Chang LC, Tsai-Morris CH, Stamenkovich D, Ferreira PC, Cohen DR, Edwards SA, Shows TB, Curran T (1988). A zinc finger-encoding gene coregulated with c-fos during growth and differentiation, and after cellular depolarization. Cell.

[24] Silverman ES, Du J, Williams AJ, Wadgaonkar R, Drazen JM, Collins T (1998). cAMP-response-element-binding-protein-binding protein (CBP) and p300 are transcriptional co-activators of early growth response factor-1 (Egr-1). Biochem J.

[25] Muller I, Rossler OG, Wittig C, Menger MD, Thiel G (2012). Critical role of Egr transcription factors in regulating insulin biosynthesis, blood glucose homeostasis, and islet size. Endocrinology.

[26] Rengarajan J, Mittelstadt PR, Mages HW, Gerth AJ, Kroczek RA, Ashwell JD, Glimcher LH (2000). Sequential involvement of NFAT and Egr transcription factors in FasL regulation. Immunity.

[27] Thiel G, Muller I, Rossler OG (2014). Expression, signaling and function of Egr transcription factors in pancreatic beta-cells and insulin-responsive tissues. Mol Cell Endocrinol.

[28] Baron V, De Gregorio G, Krones-Herzig A, Virolle T, Calogero A, Urcis R, Mercola D (2003). Inhibition of Egr-1 expression reverses transformation of prostate cancer cells in vitro and in vivo. Oncogene.

[29] Biesiada E, Razandi M, Levin ER (1996). Egr-1 activates basic fibroblast growth factor transcription. Mechanistic implications for astrocyte proliferation. J Biol Chem.

[30] Kharbanda S, Nakamura T, Stone R, Hass R, Bernstein S, Datta R, Sukhatme VP, Kufe D (1991). Expression of the early growth response 1 and 2 zinc finger genes during induction of monocytic differentiation. J Clin Invest.

[31] Suva LJ, Ernst M, Rodan GA (1991). Retinoic acid increases zif268 early gene expression in rat preosteoblastic cells. Mol Cell Biol.

[32] Nair P, Muthukkumar S, Sells SF, Han SS, Sukhatme VP, Rangnekar VM (1997). Early growth response-1-dependent apoptosis is mediated by p53. J Biol Chem.

[33] Thyss R, Virolle V, Imbert V, Peyron JF, Aberdam D, Virolle T (2005). NF-kappaB/Egr-1/Gadd45 are sequentially activated upon UVB irradiation to mediate epidermal cell death. EMBO J.

[34] Virolle T, Adamson ED, Baron V, Birle D, Mercola D, Mustelin T, de Belle I (2001). The Egr-1 transcription factor directly activates PTEN during irradiation-induced signalling. Nat Cell Biol.

[35] Hodge C, Liao J, Stofega M, Guan K, Carter-Su C, Schwartz J (1998). Growth hormone stimulates phosphorylation and activation of elk-1 and expression of c-fos, egr-1, and junB through activation of extracellular signal-regulated kinases 1 and 2. J Biol Chem.

[36] Lim CP, Jain N, Cao X (1998). Stress-induced immediate-early gene, egr-1, involves activation of p38/JNK1. Oncogene.

[37] Schwachtgen JL, Houston P, Campbell C, Sukhatme V, Braddock M (1998). Fluid shear stress activation of egr-1 transcription in cultured human endothelial and epithelial cells is mediated via the extracellular signal-related kinase 1/2 mitogen-activated protein kinase pathway. J Clin Invest.

[38] Cai Y, Liu Y, Zhang X (2006). Induction of transcription factor Egr-1 gene expression in astrocytoma cells by Murine coronavirus infection. Virology.

[39] Romagnoli L, Sariyer IK, Tung J, Feliciano M, Sawaya BE, Del Valle L, Ferrante P, Khalili K, Safak M, White MK (2008). Early growth response-1 protein is induced by JC virus infection and binds and regulates the JC virus promoter. Virology.

[40] Bedadala GR, Palem JR, Graham L, Hill JM, McFerrin HE, Hsia SC (2011). Lytic HSV-1 infection induces the multifunctional transcription factor Early Growth Response-1 (EGR-1) in rabbit corneal cells. Virol J.

[41] Morimoto K, Hooper DC, Bornhorst A, Corisdeo S, Bette M, Fu ZF, Schafer MK, Koprowski H, Weihe E, Dietzschold B (1996). Intrinsic responses to Borna disease virus infection of the central nervous system. Proc Natl Acad Sci U S A.

[42] Fu ZF, Weihe E, Zheng YM, Schafer MK, Sheng H, Corisdeo S, Raunchier FJ, Koprowski H, Dietzschold B (1993). Differential effects of rabies and borna disease viruses on immediate-early- and late-response gene expression in brain tissues. J Virol.

[43] Sakamoto KM, Nimer SD, Rosenblatt JD, Gasson JC (1992). HTLV-I and HTLV-II tax trans-activate the human EGR-1 promoter through different cis-acting sequences. Oncogene.

[44] Wagner A, Doerks A, Aboud M, Alonso A, Tokino T, Flugel RM, Lochelt M (2000). Induction of cellular genes is mediated by the Bel1 transactivator in foamy virus-infected human cells. J Virol.

[45] Chang Y, Lee HH, Chen YT, Lu J, Wu SY, Chen CW, Takada K, Tsai CH (2006). Induction of the early growth response 1 gene by Epstein-Barr virus lytic transactivator Zta. J Virol.

[46] Naranatt PP, Krishnan HH, Svojanovsky SR, Bloomer C, Mathur S, Chandran B (2004). Host gene induction and transcriptional reprogramming in Kaposi’s sarcoma-associated herpesvirus (KSHV/HHV-8)-infected endothelial, fibroblast, and B cells: insights into modulation events early during infection. Cancer Res.

[47] Tatarowicz WA, Martin CE, Pekosz AS, Madden SL, Raunchier FJ, Chiang SY, Beerman TA, Fraser NW (1997). Repression of the HSV-1 latency-associated transcript (LAT) promoter by the early growth response (EGR) proteins: involvement of a binding site immediately downstream of the TATA box. J Neurovirol.

[48] Vo N, Goodman RH (2001). CREB-binding protein and p300 in transcriptional regulation. J Biol Chem.

[49] Gwack Y, Byun H, Hwang S, Lim C, Choe J (2001). CREB-binding protein and histone deacetylase regulate the transcriptional activity of Kaposi’s sarcoma-associated herpesvirus open reading frame 50. J Virol.

[50] Kingsley-Kallesen ML, Kelly D, Rizzino A (1999). Transcriptional regulation of the transforming growth factor-beta2 promoter by cAMP-responsive element-binding protein (CREB) and activating transcription factor-1 (ATF-1) is modulated by protein kinases and the coactivators p300 and CREB-binding protein. J Biol Chem.

[51] Burysek L, Yeow WS, Lubyova B, Kellum M, Schafer SL, Huang YQ, Pitha PM (1999). Functional analysis of human herpesvirus 8-encoded viral interferon regulatory factor 1 and its association with cellular interferon regulatory factors and p300. J Virol.

[52] He F, Zhou M, Yu T, Zhao D, Zhang J, Qiu W, Lu Y, Liu Y, Wang L, Wang Y (2016). Sublytic C5b-9 triggers glomerular mesangial cell apoptosis in rat Thy-1 nephritis via Gadd45 activation mediated by Egr-1 and p300-dependent ATF3 acetylation. J Mol Cell Biol.

[53] Huang RP, Fan Y, deBelle I, Ni Z, Matheny W, Adamson ED (1998). Egr-1 inhibits apoptosis during the UV response: correlation of cell survival with Egr-1 phosphorylation. Cell Death Differ.

[54] Srivastava S, Weitzmann MN, Kimble RB, Rizzo M, Zahner M, Milbrandt J, Ross FP, Pacifici R (1998). Estrogen blocks M-CSF gene expression and osteoclast formation by regulating phosphorylation of Egr-1 and its interaction with Sp-1. J Clin Invest.

[55] Miloso M, Bertelli AA, Nicolini G, Tredici G (1999). Resveratrol-induced activation of the mitogen-activated protein kinases, ERK1 and ERK2, in human neuroblastoma SH-SY5Y cells. Neurosci Lett.

[56] Pirola L, Frojdo S (2008). Resveratrol: one molecule, many targets. IUBMB Life.

[57] Cai Q, Verma SC, Choi JY, Ma M, Robertson ES (2010). Kaposi’s sarcoma-associated herpesvirus inhibits interleukin-4-mediated STAT6 phosphorylation to regulate apoptosis and maintain latency. J Virol.

[58] Arvanitakis L, Mesri EA, Nador RG, Said JW, Asch AS, Knowles DM, Cesarman E (1996). Establishment and characterization of a primary effusion (body cavity-based) lymphoma cell line (BC-3) harboring kaposi’s sarcoma-associated herpesvirus (KSHV/HHV-8) in the absence of Epstein-Barr virus. Blood.

[59] Lo LW, Cheng JJ, Chiu JJ, Wung BS, Liu YC, Wang DL (2001). Endothelial exposure to hypoxia induces Egr-1 expression involving PKCalpha-mediated Ras/Raf-1/ERK1/2 pathway. J Cell Physiol.

[60] Pratt MA, Satkunaratnam A, Novosad DM (1998). Estrogen activates raf-1 kinase and induces expression of Egr-1 in MCF-7 breast cancer cells. Mol Cell Biochem.

[61] Xie J, Ajibade AO, Ye F, Kuhne K, Gao SJ (2008). Reactivation of Kaposi’s sarcoma-associated herpesvirus from latency requires MEK/ERK, JNK and p38 multiple mitogen-activated protein kinase pathways. Virology.

[62] Cao X, Mahendran R, Guy GR, Tan YH (1992). Protein phosphatase inhibitors induce the sustained expression of the Egr-1 gene and the hyperphosphorylation of its gene product. J Biol Chem.

[63] Miller RS, Wolfe A, He L, Radovick S, Wondisford FE (2012). CREB binding protein (CBP) activation is required for luteinizing hormone beta expression and normal fertility in mice. Mol Cell Biol.

[64] Osada S, Yamamoto H, Nishihara T, Imagawa M (1996). DNA binding specificity of the CCAAT/enhancer-binding protein transcription factor family. J Biol Chem.

[65] Zhang X, Odom DT, Koo SH, Conkright MD, Canettieri G, Best J, Chen H, Jenner R, Herbolsheimer E, Jacobsen E, Kadam S, Ecker JR, Emerson B (2005). Genome-wide analysis of cAMP-response element binding protein occupancy, phosphorylation, and target gene activation in human tissues. Proc Natl Acad Sci U S A.

[66] Chen J, Xu L, Chen S, Yang J, Jiang H (2012). Transcriptional regulation of platelet-derived growth factor-B chain by thrombin in endothelial cells: involvement of Egr-1 and CREB-binding protein. Mol Cell Biochem.

[67] Kerur N, Veettil MV, Sharma-Walia N, Sadagopan S, Bottero V, Paul AG, Chandran B (2010). Characterization of entry and infection of monocytic THP-1 cells by Kaposi’s sarcoma associated herpesvirus (KSHV): role of heparan sulfate, DC-SIGN, integrins and signaling. Virology.

[68] Gregory SM, Wang L, West JA, Dittmer DP, Damania B (2012). Latent Kaposi’s sarcoma-associated herpesvirus infection of monocytes downregulates expression of adaptive immune response costimulatory receptors and proinflammatory cytokines. J Virol.

[69] Chandran B (2010). Early events in Kaposi’s sarcoma-associated herpesvirus infection of target cells. J Virol.

[70] Wang L, Damania B (2008). Kaposi’s sarcoma-associated herpesvirus confers a survival advantage to endothelial cells. Cancer Res.

[71] Maess MB, Wittig B, Lorkowski S (2014). Highly efficient transfection of human THP-1 macrophages by nucleofection. J Vis Exp.

[72] Stuart JR, Kawai H, Tsai KK, Chuang EY, Yuan ZM (2005). c-Abl regulates early growth response protein (EGR1) in response to oxidative stress. Oncogene.

[73] Cabodi S, Morello V, Masi A, Cicchi R, Broggio C, Distefano P, Brunelli E, Silengo L, Pavone F, Arcangeli A, Turco E, Tarone G, Moro L (2009). Convergence of integrins and EGF receptor signaling via PI3K/Akt/FoxO pathway in early gene Egr-1 expression. J Cell Physiol.

[74] Veettil MV, Bandyopadhyay C, Dutta D, Chandran B (2014). Interaction of KSHV with host cell surface receptors and cell entry. Viruses.

[75] Takahashi T, Namiki Y, Ohno T (1997). Induction of the suicide HSV-TK gene by activation of the Egr-1 promoter with radioisotopes. Hum Gene Ther.

[76] Joki T, Nakamura M, Ohno T (1995). Activation of the radiosensitive EGR-1 promoter induces expression of the herpes simplex virus thymidine kinase gene and sensitivity of human glioma cells to ganciclovir. Hum Gene Ther.

[77] Bedadala GR, Pinnoji RC, Hsia SC (2007). Early growth response gene 1 (Egr-1) regulates HSV-1 ICP4 and ICP22 gene expression. Cell Res.

[78] Chen SH, Yao HW, Chen IT, Shieh B, Li C, Chen SH (2008). Suppression of transcription factor early growth response 1 reduces herpes simplex virus lethality in mice. J Clin Invest.

[79] Yao HW, Chen SH, Li C, Tung YY, Chen SH (2012). Suppression of transcription factor early growth response 1 reduces herpes simplex virus 1-induced corneal disease in mice. J Virol.

[80] Hsia SC, Graham LP, Bedadala GR, Balish MB, Chen F, Figliozzi RW (2013). Induction of Transcription Factor Early Growth Response Protein 1 during HSV-1 Infection Promotes Viral Replication in Corneal Cells. Br Microbiol Res J.

[81] Zalani S, Holley-Guthrie E, Kenney S (1995). The Zif268 cellular transcription factor activates expression of the Epstein-Barr virus immediate-early BRLF1 promoter. J Virol.

[82] Calogero A, Cuomo L, D’Onofrio M, de Grazia U, Spinsanti P, Mercola D, Faggioni A, Frati L, Adamson ED, Ragona G (1996). Expression of Egr-1 correlates with the transformed phenotype and the type of viral latency in EBV genome positive lymphoid cell lines. Oncogene.

[83] Heather J, Flower K, Isaac S, Sinclair AJ (2009). The Epstein-Barr virus lytic cycle activator Zta interacts with methylated ZRE in the promoter of host target gene egr1. J Gen Virol.

[84] Kim JH, Kim WS, Kang JH, Lim HY, Ko YH, Park C (2007). Egr-1, a new downstream molecule of Epstein-Barr virus latent membrane protein 1. FEBS Lett.

[85] Vockerodt M, Wei W, Nagy E, Prouzova Z, Schrader A, Kube D, Rowe M, Woodman CB, Murray PG (2013). Suppression of the LMP2A target gene, EGR-1, protects Hodgkin’s lymphoma cells from entry to the EBV lytic cycle. J Pathol.

[86] Ganapathi MK (1992). Okadaic acid, an inhibitor of protein phosphatases 1 and 2A, inhibits induction of acute-phase proteins by interleukin-6 alone or in combination with interleukin-1 in human hepatoma cell lines. Biochem J.

[87] Standaert ML, Bandyopadhyay G, Sajan MP, Cong L, Quon MJ, Farese RV (1999). Okadaic acid activates atypical protein kinase C (zeta/lambda) in rat and 3T3/L1 adipocytes. An apparent requirement for activation of Glut4 translocation and glucose transport. J Biol Chem.

[88] Kim YS, Ahn KH, Kim SY, Jeong JW (2009). Okadaic acid promotes angiogenesis via activation of hypoxia-inducible factor-1. Cancer Lett.

[89] Davis DA, Rinderknecht AS, Zoeteweij JP, Aoki Y, Read-Connole EL, Tosato G, Blauvelt A, Yarchoan R (2001). Hypoxia induces lytic replication of Kaposi sarcoma-associated herpesvirus. Blood.

[90] Deutsch E, Cohen A, Kazimirsky G, Dovrat S, Rubinfeld H, Brodie C, Sarid R (2004). Role of protein kinase C delta in reactivation of Kaposi’s sarcoma-associated herpesvirus. J Virol.

[91] Ohmori K, Miyazaki K, Umeda M (1998). Detection of tumor promoters by early antigen expression of EB virus in Raji cells using a fluorescence microplate-reader. Cancer Lett.

[92] Whitby D, Marshall VA, Bagni RK, Miley WJ, McCloud TG, Hines-Boykin R, Goedert JJ, Conde BA, Nagashima K, Mikovits J, Dittmer DP, Newman DJ (2007). Reactivation of Kaposi's sarcoma-associated herpesvirus by natural products from Kaposi's sarcoma endemic regions. Int J Cancer.

[93] Pan H, Xie J, Ye F, Gao SJ (2006). Modulation of Kaposi’s sarcoma-associated herpesvirus infection and replication by MEK/ERK, JNK, and p38 multiple mitogen-activated protein kinase pathways during primary infection. J Virol.

[94] Rossetto CC, Pari G (2012). KSHV PAN RNA associates with demethylases UTX and JMJD3 to activate lytic replication through a physical interaction with the virus genome. PLoS Pathog.

[95] Ye J, Shedd D, Miller G (2005). An Sp1 response element in the Kaposi’s sarcoma-associated herpesvirus open reading frame 50 promoter mediates lytic cycle induction by butyrate. J Virol.

[96] Sakakibara S, Ueda K, Chen J, Okuno T, Yamanishi K (2001). Octamer-binding sequence is a key element for the autoregulation of Kaposi’s sarcoma-associated herpesvirus ORF50/Lyta gene expression. J Virol.

[97] Bottero V, Sharma-Walia N, Kerur N, Paul AG, Sadagopan S, Cannon M, Chandran B (2009). Kaposi sarcoma-associated herpes virus (KSHV) G protein-coupled receptor (vGPCR) activates the ORF50 lytic switch promoter: a potential positive feedback loop for sustained ORF50 gene expression. Virology.

[98] Kati S, Hage E, Mynarek M, Ganzenmueller T, Indenbirken D, Grundhoff A, Schulz TF (2015). Generation of high-titre virus stocks using BrK.219, a B-cell line infected stably with recombinant Kaposi’s sarcoma-associated herpesvirus. J Virol Methods.

[99] Thakker S, Purushothaman P, Gupta N, Challa S, Cai Q, Verma SC (2015). Kaposi’s Sarcoma-Associated Herpesvirus Latency-Associated Nuclear Antigen Inhibits Major Histocompatibility Complex Class II Expression by Disrupting Enhanceosome Assembly through Binding with the Regulatory Factor X Complex. J Virol.

[100] King CA, Li X, Barbachano-Guerrero A, Bhaduri-McIntosh S (2015). STAT3 Regulates Lytic Activation of Kaposi’s Sarcoma-Associated Herpesvirus. J Virol.

[101] Strober W (2015). Trypan Blue Exclusion Test of Cell Viability. Curr Protoc Immunol.

